# Haplotype-resolved genome of *Agastache rugosa* (Huo Xiang) provides insight into monoterpenoid biosynthesis and gene cluster evolution

**DOI:** 10.1093/hr/uhaf034

**Published:** 2025-02-01

**Authors:** Chanchan Liu, DiShuai Li, Jingjie Dang, Juan Shu, Samuel J Smit, QiNan Wu, Benjamin R Lichman

**Affiliations:** Jiangsu Collaborative Innovation Center of Chinese Medicinal Resources Industrialization, Nanjing University of Chinese Medicine, Nanjing 210023, China; College of Pharmacy, Nanjing University of Chinese Medicine, Nanjing 210023, China; Jiangsu Collaborative Innovation Center of Chinese Medicinal Resources Industrialization, Nanjing University of Chinese Medicine, Nanjing 210023, China; College of Pharmacy, Nanjing University of Chinese Medicine, Nanjing 210023, China; Jiangsu Collaborative Innovation Center of Chinese Medicinal Resources Industrialization, Nanjing University of Chinese Medicine, Nanjing 210023, China; College of Pharmacy, Nanjing University of Chinese Medicine, Nanjing 210023, China; Jiangsu Collaborative Innovation Center of Chinese Medicinal Resources Industrialization, Nanjing University of Chinese Medicine, Nanjing 210023, China; College of Pharmacy, Nanjing University of Chinese Medicine, Nanjing 210023, China; Department of Biology, Centre for Novel Agricultural Products, University of York, York, YO10 5DD, UK; Jiangsu Collaborative Innovation Center of Chinese Medicinal Resources Industrialization, Nanjing University of Chinese Medicine, Nanjing 210023, China; College of Pharmacy, Nanjing University of Chinese Medicine, Nanjing 210023, China; Department of Biology, Centre for Novel Agricultural Products, University of York, York, YO10 5DD, UK

## Abstract

Monoterpenoids are small volatile molecules produced by many plants that have applications in consumer products and healthcare. Plants from the mint family (Lamiaceae) are prodigious producers of monoterpenoids, including a chemotype of *Agastache rugosa* (Huo Xiang), which produces pulegone and isomenthone. We sequenced, assembled and annotated a haplotype-resolved chromosome-scale genome assembly of *A. rugosa* with a monoterpene chemotype. This genome assembly revealed that pulegone biosynthesis genes are in a biosynthetic gene cluster, which shares a common origin with the pulegone gene cluster in *Schizonepeta tenuifolia*. Using phylogenetics and synteny analysis, we describe how the clusters in these two species diverged through inversions and duplications. Using Hi-C analysis, we identified tentative evidence of contact between the pulegone gene cluster and an array of pulegone reductases, with both regions also enriched in retrotransposons. This genome and its analysis add valuable and novel insights to the organization and evolution of terpenoid biosynthesis in Lamiaceae.

## Introduction

Volatile monoterpenoids are widespread plant specialized metabolites with various applications including flavors and fragrances, insect repellents [1] and medicinal treatments [2]. Plants from the Nepetoideae clade of the mint family (Lamiaceae) are prodigious producers of bioactive monoterpenoids [3], including *Mentha* spp. (e.g. menthol) [4] and *Nepeta* spp. (e.g. nepetalactone) [5, 6]. Two closely related plants in this clade—*Agastache rugosa* (Fisch. & C.A.Mey.) Kuntze and *Schizonepeta tenuifolia* (Benth.) Briq.—are known to produce pulegone, a monoterpenoid with carcinogenic [7], psychoactive [8] and antihistamine properties [9]. These two plants are used in traditional Chinese medicine, named Huo Xiang ($\raisebox{-1pt}{\includegraphics{\bwartpath uhaf034fx1}}$) and Jing Jie ($\raisebox{-1pt}{\includegraphics{\bwartpath uhaf034fx2}}$) respectively.

Monoterpenoids are derived from geranyl pyrophosphate through the action of a terpene synthase (TPS), with different TPSs leading to different monoterpenoid skeletons [10]. This scaffold forming step typically occurs in the plastid, with the precursors derived from the plastidal 2-C-methyl-D-erythritol 4-phosphate pathway [11]. Downstream modification of terpenes occurs through the action of enzymes such as cytochrome P450s [12]. Genes encoding enzymes involved in terpenoid biosynthesis pathways can sometimes be found in close genomic proximity in biosynthetic gene clusters (BGCs) [13, 14].

BGCs are typically defined as three or more non-homologous genes acting in a single biosynthetic pathway in close genomic proximity [14]. Well studied examples include those involved in alkaloid biosynthesis in poppy [15], in triterpenoid biosynthesis in oat [16] and diterpenoid biosynthesis in rice [17]. Notable advances in the field include the identification of transcription factors controlling BGCs in tomato phenolamide biosynthesis [18], and of chromosomal interactions within [19, 20] and between [21] BGCs. In Lamiaceae, a conserved diterpenoid cluster has been characterized in multiple species [22, 23], and an iridoid monoterpenoid BGC has been found determined to have emerged within the *Nepeta* lineage [5].

**Figure 1:**
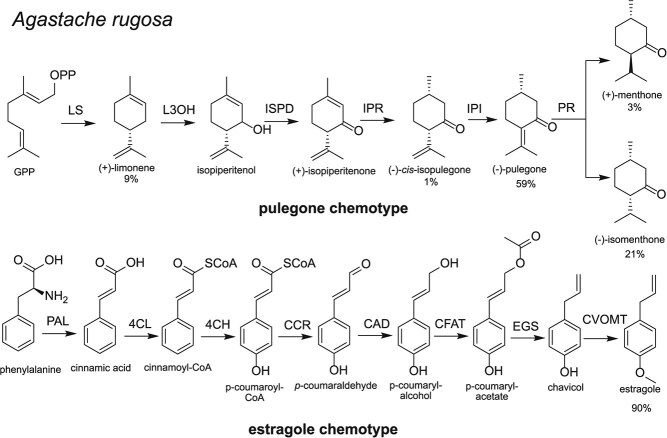
Biosynthetic pathways of major products in *Agastache rugosa* chemotypes. The menthone biosynthesis pathway: limonene synthase (LS); limonene 3-hydroxylase (L3OH); isopiperitenol dehydrogenase (ISPD); isopipertenone reductase (IPR); isopulegone isomerase (IPI, not yet discovered); pulegone reductase (PR). The estragole biosynthesis pathway: phenylalanine ammonia-lyase (PAL); 4-coumarate:co-enzyme A ligase (4CL); cinnamate 4-hydroxylase (4CH); cinnamoyl-CoA reductase (CCR); cinnamyl alcohol dehydrogenase (CAD); caffeoyl-CoA transferase (CFAT); eugenol synthase (EGS); chavicol O-methyl transferase (CVOMT).

We recently examined the genomic basis of pulegone biosynthesis in *S. tenuifolia* and discovered a BGC that contained genes involved in pulegone biosynthesis [24]. The biosynthesis of pulegone, and related compounds, is now resolved, except for the theoretical isopulegone isomerase (IPI), which is yet to be identified (Fig. 1). The pulegone BGC has an unusual ‘bipartite’ structure with mirrored biosynthetic regions separated by 18 genes. Through phylogenomic analysis we determined that, compared to *Nepeta* spp., *Hyssopus officinalis* [5] and *Mentha longifolia* [4], the BGC formed within the *S. tenuifolia* lineage through insertion of pathway genes into a TPS-rich region, followed by an inverted duplication rearrangement.

The closely related plant *A. rugosa* is known to have at least two chemotypes: the pulegone type and the estragole type, which accumulate monoterpenoids and phenylpropanoids respectively (Fig. 1) [25]. We recently examined these chemotypes and discovered that, in the pulegone chemotype, monoterpenoid genes were upregulated, and phenylpropanoid genes were downregulated [25]. We also determined that isomenthone accumulates in older leaves at the expense of pulegone, which correlates with increased expression of pulegone reductases [26].

In this work, we set out to sequence the genome of a pulegone producing *A. rugosa*. This would allow us to identify the genomic basis of pulegone production and compare this with the closely related *S. tenuifolia*. Through this work, we discovered that a syntenic pulegone BGC is present in *A. rugosa* but crucially does not show evidence of an inversion and appears to have lineage specific gene duplication events. We also leverage HiC-data to identify three dimensional interactions with the BGC including tentative evidence of interchromosomal interactions between biosynthetic genes.

## Results

### Sequencing, assembly, and annotation of the pulegone chemotype *Agastache rugosa* genome

We set out to sequence the genome of an *Agastache rugosa* plant with a pulegone chemotype (Fig. 1). To do this, we identified a pulegone producing individual based on our previous metabolite analysis work [25]. From this individual, we extracted high-molecular weight DNA, and then conducted HiFi sequencing using the PacBio Sequel II platform, generating 31.82 Gb of effective data from one cell (Supplementary Table S1). Subsequently, we constructed a Hi-C standard library using the DNBseq platform, obtaining 138.14 Gb of clean data (Supplementary Table S2). Genome assembly was performed using Hifiasm 3, which integrated HiFi and Hi-C data from paired-end sequencing. This preliminary assembly yielded two sets of haploid genome contig sequences, termed Hap1 and Hap2 (Supplementary Table S3). The total lengths of contig-level assemblies for Hap1 and Hap2 were 487 162 419 bp and 468 104 839 bp, respectively. After Hi-C scaffolding with Juicer and 3D-DNA software, 451 737 736 bp and 454 358 246 bp of sequences were localized to nine chromosomes for Hap1 and Hap2, respectively, resulting in Hi-C scaffolding rates of 92.73% and 97.06% (Supplementary Table S4). The GC content ratios and total lengths were similar for both haplotypes, indicating good reproducibility. Additionally, the completeness of the genome assemblies at the contig level was 97.29% and 97.64% respectively, as measured by Complete BUSCOs, and these values were maintained at the chromosomal level (Supplementary Table S5). The assembled genome size was in line with k-mer analysis of unassembled reads which estimated a haplotype genome size of 461 Mb with 0.4% heterozygosity (Supplementary Fig. S1).

We annotated the genome assembly with transposable elements (TEs) and repeats. After comparison with the RepBase database (http://www.girinst.org/repbase), 60.88% and 62.20% of repetitive sequences were identified in Hap1 and Hap2, respectively (Supplementary Tables S6 and S7). In these genomes, LTR (long terminal repeat) retrotransposons, particularly Gypsy and Copia subtypes and other unclassified LTR transposons, comprised a significant proportion. Annotation of Hap1 and Hap2 using homology predictions based on data from six related species (*A. thaliana, N. cataria, P. citriodora, P. frutescens, S. hispanica, T. grandis*) yielded 31 334 and 31 578 genes, respectively (Supplementary Table S8). The completeness of the sequenced genome was evaluated using BUSCO, revealing a high level of completeness (>95%) (Supplementary Table S5).

Whilst we were in the process of analyzing this new genome, another genome of *A. rugosa* was published by Park *et al* [27]. The method of sequencing differed to our use of PacBio + Hi-C, with Park *et al* using ONT nanopore R9.4.1, polished with Illumina and scaffolded with Hi-C. Although both are high-quality genomes, scaffolded to chromosome level and near complete according to BUSCO, a difference lies in that we present a haplotype-resolved assembly. Furthermore, there is a major difference in the contig level contiguity, with our contig N50 (48 Mb, hap2) only marginally below scaffold N50 (59 Mb, hap2) (Supplementary Table S3) whereas in the Park *et al* genome the contig assembly is more fragmented (contig N50 of 4 Mb compared to scaffold N50 of 52 Mb). This could have consequences for the arrangement and orientation of contigs on the chromosome-level scaffold, which does appear to be different across the two assemblies (Supplementary Fig. S2A).

Furthermore, we assessed the Park *et al* genome gene models for genes involved in pulegone biosynthesis but could not identify all expected genes. Using standard BLAST analysis, we found expected homologs of limonene synthase (LS) [28], isopiperitenone reductase (IPR) and isopiperitenone dehydrogenase (ISPD) but we did not find a limonene 3-hydroxylase (L3OH) homolog, despite it being recently characterized from the plant [25]. Finally, we noted that the Hi-C depth was notably lower than in our genome and insufficient to perform downstream assessment (see below) (Supplementary Fig. S2B). All these factors support the value of our new haplotype-resolved *A. rugosa* assembly.

**Figure 2:**
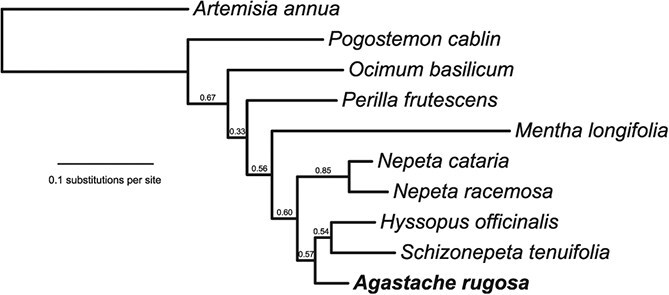
Species tree. Whole-genome based phylogeny of mint family species, inferred using OrthoFinder v2.5.4 with default parameters [DIAMOND for sequence similarity searches, MCL for clustering, STAG (Species Tree Inference from All Genes) and STRIDE (Species Tree Root Inference from Gene Duplication Events) for species tree inference]. Numbers on branches refer to STAG bipartition proportions (the proportion of input trees in which that bipartition occurs) as determined by OrthoFinder. *Artemisia annua* used as an outgroup.

To place the species in context, we inferred a species tree with selected genomes [4, 5, 24, 29–32] using OrthoFinder [33] (Fig. 2). This analysis recovered the expected Nepetinae clade featuring *A. rugosa*, *Nepeta* spp., *Hyssopus officinalis* and *S. tenuifolia*. Within this subtribe, the *Nepeta* clade is sister to the other species lineages, and *A. rugosa* is sister to *H. officinalis* and *S. tenuifolia*. This is in line with other comprehensive phylogenetic analyses [34–36].

### The pulegone biosynthesis pathway is clustered

*Agastache rugosa* is notable for its production of volatile compounds, which are differentially produced across chemotypes (Fig. 1) [25]. One major chemotype consists predominantly of monoterpenoids including pulegone, whereas the other major chemotype is mostly estragole, which is a phenylpropanoid [37]. We previously assessed gene expression of the biosynthetic pathways across [25]. With the genome available, we were able to identify the location of the biosynthetic genes. In particular, we were interested in the clustering of genes into BGCs, which can provide insight into the regulation, evolution and composition of pathways [13]. We focused just on Hap 2 for this analysis, as it had marginally better N50 and BUSCO complete scores than Hap 1 (Supplementary Tables S3 and S5). Alongside identifying the location of biosynthetic genes, we inferred gene trees to assess their orthology and evolution.

**Figure 3:**
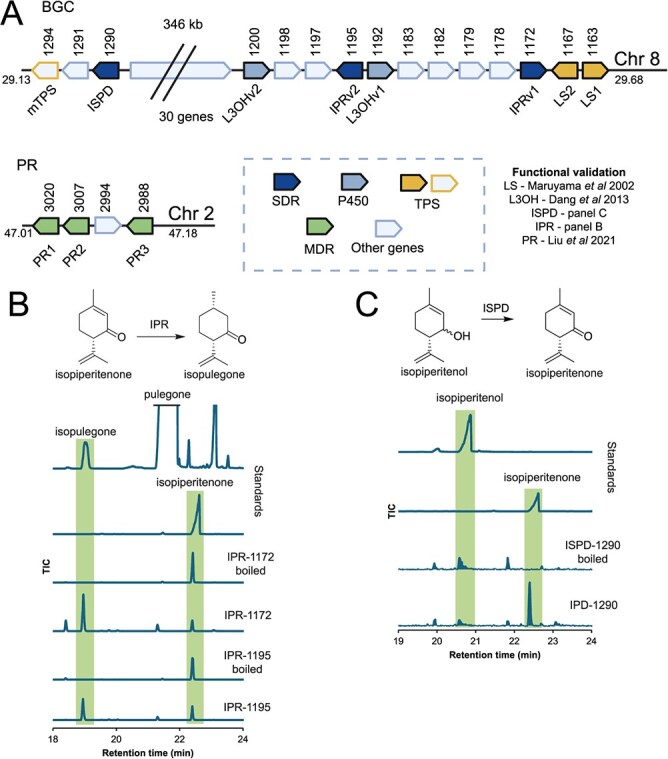
Pulegone biosynthesis genes. (A) The genomic context of pulegone biosynthetic genes. The BGC region is responsible for pulegone biosynthesis and exists as a core cluster with duplicated LS, IPR and L3OH, with ISPD over 345 kb away. The PR genes can be found as an array on chromosome 2. The number above the gene represents the gene number (chromosomes numbered separately). Gene length and intergenic space is normalized for depiction. Box shows color categories for gene types. (B) *In vitro* activity of A. rugosa IPRs. Recombinantly expressed IPRs were incubated with isopiperitenone and NADPH (with co-factor recycling) and shown to form isopulegone based on minor peak in pulegone standard [1]. Peak heights of standards scaled for depiction. IPR-1172 = IPRv1 and IPR-1195 = IPRv2. EI spectra shown in Supplementary Fig. S4B. (C) *In vitro* activity of A. rugosa ISPD. Recombinantly expressed ISPD were incubated with isopiperitenol and NAD (with co-factor recycling) and shown to form isopiperitenone. Peak heights of standards scaled for depiction. EI spectra shown in Supplementary Fig. S6B.

We first identified limonene synthase (LS), the first step in the pulegone biosynthesis pathway, which had previously been characterized from *A. rugosa* [28]. We found a region in chromosome 8 containing two copies of LS. These paralogs (LS1, h2tig37.1163 and LS2, h2tig37.1167) are identical at the nucleotide level and in a tail-to-tail orientation (Fig. 3). Phylogenetic analysis indicates the *A. rugosa* LSs are orthologous to those from *S. tenuifolia* (Supplementary Fig. S3).

Adjacent to the LSs is a gene (IPRv1, h2tig37.1172) with 92% identity to the isopiperitenone reductases (IPR), recently characterized from *S. tenuifolia* [24]. Five genes upstream (85 kb), there is a second copy of the putative IPR (IPRv2, h2tig37.1195, 99% identity to h2tig37.1172). Phylogenetic analysis suggested that these are orthologs of *S. tenuifolia* IPR and therefore likely to have the same function (Supplementary Fig. S3A). Indeed, through recombinant expression in *E. coli* we were able to show that the encoded enzymes could catalyze the reduction of isopiperitenone to isopulegone (Fig. 3B). IPRv2 is adjacent to a gene (L3OHv1, h2tig37.1195) which is orthologous to *S. tenuifolia* limonene 3-hydroxylase (Supplementary Fig. S5). An identical copy is also present nearby (L3OHv2, h2tig37.1200). The activity of this *A. rugosa* L3OH was recently verified [25].

An ISPD ortholog (evm.model.h2tig37.1290, Supplementary Fig. S6) is present 346 kb away. We verified the activity of the encoded ISPD through recombinant expression in *E. coli*, showing it could oxidize isopiperitenol into isopiperitenone (Fig. 3C). The ISPD gene is close to an uncharacterized monoterpene synthase (evm.model.h2tig37.1294) which is 79% identical on the nucleotide level to LS1 and LS2 but not directly orthologous (Supplementary Fig. S3).

Overall, the region containing duplicate copies of LS, L3OH and IPR is a BGC, defined by the proximity of non-homologous sequences functioning in a biosynthetic pathway [14]. The LS and L3OH genes in this BGC have previously been characterized [25, 28], and the IPR and ISPD have been characterized here (Supplementary Figs S4 and S6) This BGC is present in both haplotypes (Supplementary Fig. S7). It is reminiscent of the bipartite cluster for pulegone biosynthesis in closely related *S. tenuifolia* [24].

The TE content of the cluster was also assessed (Supplementary Fig. S8), with peaks in TE density close to ISPD, between the LS paralogs and in the region containing duplicated IPRs and L3OHs. Indeed, the core cluster region (containing LS, L3OH and IPR paralogs) was enriched in LTR annotations (70% compared to 57% genome wide), especially LTR/Gypsy/Athila (10% vs 3% genome wide) (Supplementary Table S9). The ISPD region was enriched in LTRs of unknown class (34% compared to 23% genome wide).

Akin to *S. tenuifolia*, genes encoding pulegone reductases (PRs) were not present in the BGC region but elsewhere in the genome as tandem repeats (Fig. 3A). We previously characterized *A. rugosa* PR [38]. On chromosome 2, we found three copies of genes encoding this enzyme (PR1–3, 99% amino acid identity) arranged in a tandem array (Fig. 3).

We also investigated the location of the estragole pathway genes, and found the genes typically as single copy genes, dispersed across the genome (Supplementary Fig. S9A). An exception to this was the CCR gene, encoding the aldehyde forming cinnamoyl-CoA reductase, which had four tandem copies on chromosome 9. Curiously, the estragole pathway genes, previously shown to be downregulated in this chemotype accession [25], did not appear to have coordinated expression across tissues (Supplementary Fig. S9B), which may contribute to relatively low quantities of estragole produced.

### Synteny analysis shows variation in BGC region across Nepetinae

**Figure 4:**
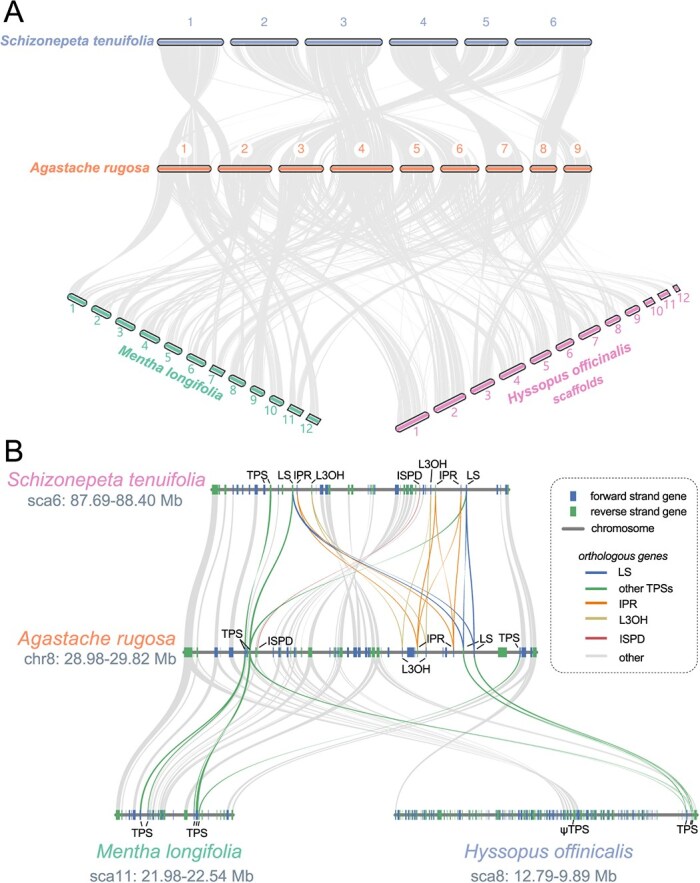
Synteny analysis. (A) Macrosynteny analysis showing large scale genomic differences between closely related species. All genome assemblies are at a chromosome scale except for H. officinalis which is at scaffold scale. (B) Collinearity (synteny) analysis of bipartite BGC compared with other high-quality Lamiaceae genomes. Lines indicate orthologous genes (see legend inset). LS and TPS connections have been drawn based on inferred clade relationships (Supplementary Fig. S3).

Next, we conducted synteny analysis, comparing the *A. rugosa* genome structure with the closely related genomes (*S. tenuifolia*, *A. rugosa*, *M. longifolia* and *H. officinalis*) (Fig. 4). The macrosynteny analysis (Fig. 4A) detailed large scale chromosomal rearrangements, with the *S. tenuifolia* genome showing the fewest differences compared to the *A. rugosa* assembly.

We used microsynteny analysis to compare the *A. rugosa* BGC to that in *S. tenuifolia* (Fig. 4B) [24]. Indeed, the clusters are syntenic, which, taken together with the orthologous relation of the gene content (Supplementary Figs S3–S6), implies that clusters share a common origin. The gene content is similar, each having one copy of ISPD, duplicates of LS and L3OH, and two or more copies of IPR; both BGCs are split into two distinct regions separated by a multi-megabase region of non-biosynthetic genes. However, there are clear differences between the two clusters. For example, in *S. tenuifolia*, the region is flanked by the two near-identical paralogous LSs, yet in *A. rugosa* the 5’-TPS (h2tif37.1294) is more diverged (Supplementary Fig. S3). In *S. tenuifolia*, the LS, IPR, and L3OH gene duplicates are spread across both parts of the cluster, whereas in *A. rugosa* they are all in the 3′ region. Furthermore, it is notable that the higher similarity of duplicate paralogs compared to orthologs indicates that that BGC gene duplications occurred independently in the *S. tenuifolia* and *A. rugosa* lineage (Supplementary Figs S3, S5, and  S6).

Most notably, the core gene cluster region is inverted between *A. rugosa* and *S. tenuifolia.* Based on comparison with *M. longifolia* it appears that *A. rugosa* represents the non-inverted structure and an inversion has occurred in the *S. tenuifolia* lineage (Fig. 4B) [24]. This implies that genes entered the region in the common ancestor of *H. officinalis*, *A. rugosa* and *S. tenuifolia* and, in the latter lineage an inversion occurred, with the TPSs representing an approximate boundary. The lack of a BGC in hyssop likely represents a loss, perhaps related to the presence of a non-syntenic region in this locus.

### Intrachromosomal interactions with BGC

There is increasing interest in the 3D arrangement of genomes, including work related to plant BGCs, where 3D genomics can lead to gene discovery [20] or insight into regulation [19, 39]. We have recently proposed a theory that suggests the 3D contacts in a genome may be a mediator of gene movement [13]. We therefore set out to understand the interactions between the BGC and the rest of the genome.

**Figure 5:**
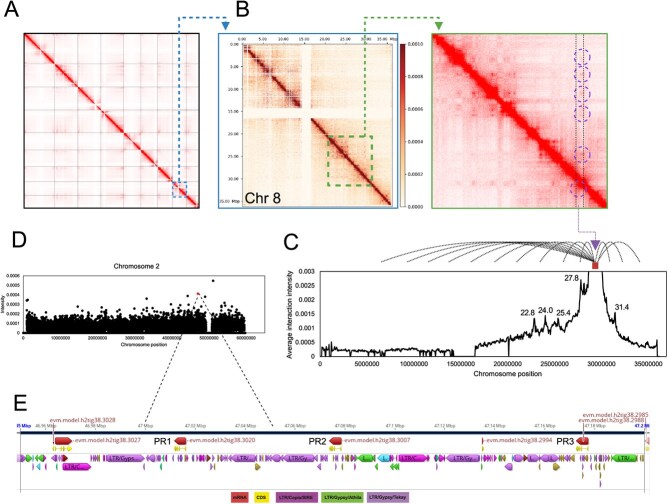
Three dimensional BGC interactions from Hi-C interaction data. (A) Genome-wide level mapping of gene interactions. (B) Chromosome 8 Hi-C interactions with cluster region and interactions highlighted. (C) Average interaction intensity of gene cluster at 29.1 Mb–29.7 Mb with chromosome 8 at 50 kbp resolution. Annotations in interacting regions can be found in Supplementary Fig. S9. (D) Interaction intensities of gene cluster (29.1 Mb–29.7 Mb) with chromosome 2. The highlighted point represents interaction with region containing PR1 (47.00–47.05 Mbp, interaction is significantly greater than chromosome average, *P* = .0002). (E) Pulegone reductase array region showing BGC interacting region and gene and TE annotations (from *de novo* RepeatMasker).

To do this, we repurposed Hi-C data, collected originally to aid genome assembly. We had data from leaf at 100x coverage, which was of sufficient depth to analyze the data to 50 kbp resolution. The whole-genome interaction heatmaps revealed that Hi-C assembled chromosomes show high interaction intensity between adjacent sequences (diagonal positions), whereas non-adjacent sequences (off-diagonal positions) display weak interaction signals. This distribution aligns with the principles of Hi-C-assisted genome assembly, substantiating the assembly’s effectiveness (Fig. 5A).

First, we looked into the 3D organization of chromosome 8, looking into A/B compartmentalization (euchromatin/heterochromatin), topologically associated domains (TAD), and loop structures (Supplementary Fig. S10). It was found that the gene cluster region has consistently lower absolute values of PC1, suggesting that the whole region was in a single chromatin state. Outside of the BGC PC1 values were higher, indicating a different chromatin state. The 3′ part of the cluster, containing copies of LS, L3OH and IPR, is arranged in a TAD region, as supported in the gene interaction heatmap with increased local interactions. The region with ISPD does not appear to be in the same TAD. We also observed a loop connecting the BGC region to a nearby region (2.4 Mb away) that contains uncharacterized glucosyltransferases.

Through the analysis of the Chr 8 gene interaction heatmap, it was observed that the gene cluster region 29.1–29.7 Mb shows some distinct interactions with other regions within the chromosome (Fig. 5B). Consequently, we were able to pinpoint their exact chromosomal locations (Fig. 5C). Upon further investigation of the precise locations of these most intense interactions with BGC, we discovered they were regions enriched in LTRs (71% vs 57% genome wide) including LTR/Copia/SIRE (23% vs 14% genome wide) and LTR/Gypsy/Athila (5% vs 3% genome wide) retrotransposons (Supplementary Fig. S11, Supplementary Table S9).

### Interchromosomal interactions with BGC

Next, we looked for interchromosomal interactions with the BGC. To do this, we collected interaction data from the BGC region (chromosome 8: 27.1–27.9 Mbp) and the whole genome (Supplementary Fig. S12A). To screen for regions with most intense interactions with the BGC we normalized the interactions within each chromosome (Supplementary Fig. S12B), and then looked for values significantly diverse from the mean, assuming a normal distribution and correcting for multiple tests (Supplementary Fig. S12C). This revealed regions with slightly increased LTR content compared to the rest of the genome (60% compared to 57%) (Supplementary Table S9).

We then specifically looked at the interaction between the pulegone BGC and the PRs, which encode enzymes that reduce pulegone to menthone. To our surprise there was a significant association between the BGC and the region containing a PR1 (chromosome 2, 47.00 Mb – 47.05 Mb) (Fig. 5D). Whilst this was not significant in the genome wide analysis due to multiple test correction (i.e. Supplementary Fig. S12), it is the second most intense BGC interacting region of chromosome 2, and in a targeted assessment this is significant compared to the average chromosomal interactions (*P* = .0002). The PR array region is highly enriched in LTRs (78% compared to 60% genome wide), especially those annotated as LTR/Gypsy which are found across 59% of bases in the array compared to only 18% genome wide (Fig. 5E, Supplementary Table S9). This interaction, albeit only detected in a single dataset, represents a hint that there may be interchromosomal 3D clustering of biosynthetic genes. Such a phenomenon has previously been described with long-distance interactions between BGCs and tandem arrays [21]. We are planning to conduct further experiments to verify this interaction in *A. rugosa*.

## Discussion

We set out to investigate the genomic basis for monoterpenoid biosynthesis in *A. rugosa*. Most notably, we discovered a BGC containing genes involved in pulegone biosynthesis (Fig. 3). This was syntenic to a BGC we recently described in the closely related pulegone producing *S. tenuifolia* (Fig. 4). Based on this syntenic and supporting phylogenetic analysis, which shows orthologous relationships of BGC genes in the two species (Supplementary Figs S3–S6), it appears that the BGC was formed in a common ancestor of *S. tenuifolia* and *A. rugosa* in a region rich in limonene synthase-like TPSs [24]. The absence of the BGC in *H. officinalis* (Fig. 4), which shares the same common ancestor with the two species (Fig. 2), indicates a loss that may be associated with an insertion in that region and/or a more complex evolutionary history (e.g. introgression or incomplete lineage sorting).

**Figure 6:**
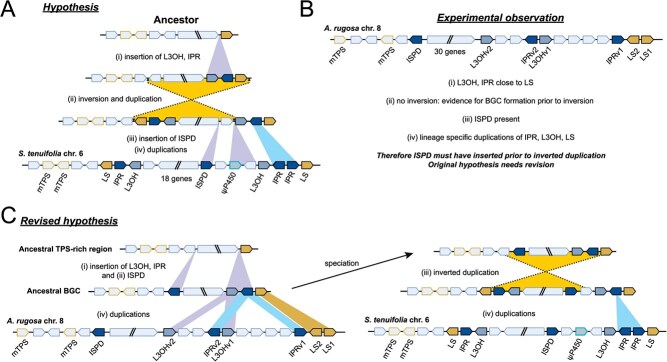
Evolutionary origin of the pulegone BGC. (A) Original hypothesis of BGC formation based on S. tenuifolia genome assembly. (B) Observation of the A. rugosa BGC and consequences for the hypothesis. (C) Revised hypothesis of BGC formation with new data considered.

We previously hypothesized that BGC formation in the *S. tenuifolia* lineage occurred first (i) by insertion of L3OH and IPR adjacent to LS, followed by (ii) an inverted duplication, then (iii) insertion of ISPD into one part of the BGC and finally (iv) recent duplications (Fig. 6A) [24]. The new observation of the *A. rugosa* BGC provides a test of this hypothesis: (i) is validated by the syntenic and orthologous L3OH and IPR next to LS, (ii) is verified by the lack of an inversion in *A. rugosa*, supporting BGC formation prior to inversion (Fig. 6B). There are also (iv) recent duplications of LS, IPR and L3OH, which, based on phylogenetics were specific to the *A. rugosa* linage (Supplementary Figs S3–S6).

The presence of ISPD adjacent to a TPS in *A. rugosa*, away from the main BGC, however, does not support part (iii) of the original hypothesis as ISPD is present despite no inversion. Instead, the ISPD must have been inserted prior to the inversion in the *S. tenuifolia* lineage. This process, testing our evolutionary hypothesis using new genomic observations, has allowed us to draw up a revised hypothesis for how the BGC evolved and diversified (Fig. 6C).

The validation here that the pulegone BGC formed prior to inversion in the *S. tenuifolia* lineage lends support to the concept that inversion events may play important roles in BGCs [40] and other ‘concentrated genetic architectures’ like supergenes [41]. An inversion can repress recombination, linking functionally related genes in proximity and ensuring inheritance as a functional cluster [42]. The inversion also served to bring the ISPD closer to the other genes, creating a more compact cluster. The mechanistic connection between the inversion and duplication of the LS-L3OH-IPR genes remains unresolved. The exact boundaries of the inversion have not been confirmed but the border is approximately at the TPS/LS1 in *A. rugosa*.

We have recently developed a theory for BGC formation that, at its heart, requires 3D contact between regions containing functionally related biosynthetic genes [13]. We leveraged Hi-C data originally collected for genome scaffolding to investigate this hypothesis. On an intrachromosomal and genome-wide level we identified regions that the BGC appeared to have significant interactions with (Supplementary Figs S10–S12). A targeted search of interactions between the BGC and the related PR genes revealed a significant interchromosomal interaction between the BGC and region containing PR1 (*P* = .0002, Fig. 5D and E). It is notable that the product of PR, isomenthone, is the major monoterpenoid in older leaves, so the BGC and PR have a close functional relationship in leaves, which is the origin of the HiC sample. Whilst this is a tantalizing hint of clustering across three dimensions, we acknowledge this is only a single datapoint. Investigations into long range chromatin loops (>100 kb and interchromosomal) in arabidopsis, soybean and rice found that loop anchors significantly overlap with BGCs and tandem arrays, indicating interactions between BGCs and tandem arrays on a genome-wide scale [21]. These loop anchors were also found to be closely associated with H3K27me3 epigenetic markers, which are known to be associated with silent BGCs [31]. Based on this we plan to examine the BGC and array interaction further by collecting Hi-C data from different tissues alongside analysis of epigenetic markers.

Furthermore, our analysis has highlighted regions involved in monoterpenoid biosynthesis (i.e. the BGC and PR array) are enriched in transposable elements, especially different LTR classes (Supplementary Table S9). The association of TEs with BGCs is well known [13, 14]. Furthermore, there is emerging evidence of TE involvement in genome organization [43], which we suspect can play a role in BGC formation [13].

## Conclusion

Overall, through the sequencing of the *A. rugosa* genome plus phylogenomic analysis, we have gained considerable insight into monoterpenoid biosynthesis in the mint family with observations on cluster organization and evolution that may have wider implications across research into plant specialized metabolism.

## Materials and methods

### Plant material

The seeds of *Agastache rugosa* used for genome sequencing were collected from the Medicinal Garden in the Nanjing University of Chinese medicine. Then the seeds were grown in a growth incubator under 10 000 lx intensity and 50% humidity with 16/8 h light/dark photoperiod at 25°C. Plant tissues were carefully removed, immediately snap-frozen in liquid nitrogen, and stored at 80°C for RNA and DNA extraction.

### DNA extraction

To process tissue samples, we used the MGIEasy Plant Genome DNA extraction kit (BGI, Shenzhen, Guangdong, China). Approximately 0.3–3 g was ground into a powder with liquid nitrogen and mixed with 3 ml of Tissue Lysis Buffer 1 in a 5 ml centrifuge tube, followed by incubation at 65°C for 30 minutes. After low-temperature centrifugation for 10 minutes, the supernatant was transferred to a new tube, and 1 ml of Settling Buffer 2 was added, followed by another low-temperature centrifugation. The supernatant was then moved to 2 ml centrifuge tubes, where 900 μl of lysed supernatant, 900 μl of DNA Binding Buffer, and 50 μl of Magnetic-Bead Buffer were added; this mixture was gently mixed and allowed to sit at room temperature for 5 minutes for DNA binding. The tubes were then placed in a magnetic separator until the beads are absorbed and the supernatant was clear, after which the supernatant was discarded, and the beads washed twice with 75% v/v ethanol and dried. An 80–300 μl preheated Elution Buffer (50°C) was added to the beads, which were then resuspended and allowed to sit before being placed in the magnetic separator again. Finally, the clear supernatant was transferred to new 1.5 ml centrifuge tubes and stored at 4°C for nucleic acid quality control.

### Genome sequencing and assembly

High-fidelity (HiFi) sequencing was conducted utilizing the PacBio Sequel II platform (PacBio, San Diego, CA, USA). High-quality, long genomic DNA fragments were initially extracted utilizing the magnetic bead method, followed by fragmentation using a Megaruptor system and subsequent size selection of 13–16 kb fragments via Sage ELF (Sage Science, Beverly, MA, USA). Utilizing the SMRTbell Express Template Prep 2.0 kit (PacBio, San Diego, CA, USA), these DNA segments were then affixed with hairpin-structured, circular single-stranded PacBio adapters at both extremities, resulting in the formation of a ‘dumbbell-shaped’ SMRTbell library. The circular consensus sequencing algorithm (v4.0.0) was employed to generate high-quality, high-accuracy reads from multiple passes of a single SMRTbell template (accuracy rate ≥ 99%).

For chromatin conformation capture analyses, genomic DNA underwent enzymatic digestion with MboI to construct a standard Hi-C library. This library was subsequently sequenced using the DNBseq platform (BGI, Shenzhen, Guangdong, China). The initial haplotype-resolved genomic assembly was efficiently conducted using Hifiasm3 (v1.61) [44], integrating both HiFi and paired-end Hi-C sequencing data with the software’s default parameters to optimize the assembly process.

### Hi-C assisted assembly

Alignment and evaluation of the Hi-C library were performed using Juicer (v1.6) [45], which mapped paired-end sequencing reads to the assembled genome. This process quantified the proportion of unique Hi-C contacts mapped to the assembled genome. Contig orientation and clustering, based on Hi-C linkage signals, were achieved using both Juicer (−s MboI) and 3D-DNA (v180419; −r 2) software [46], aiding in the scaffolding of contigs across nine chromosomes.

After Hi-C-assisted assembly, the integrity of the assembled genome was evaluated employing the Benchmarking Universal Single-Copy Orthologues (BUSCO; v5.1.2) [47] eudicots_odb10 database (−m genome -l eudicots_odb10), which determined the assembly’s completeness and redundancy by identifying the prevalence of conserved single-copy orthologs among species.

### Genome annotation

Repetitive sequences in the *Agastache rugosa* genome were annotated through homology-based and *de novo* prediction approaches. Through the RepBase repository [48], homology-based annotations were conducted to discern sequences akin to established repetitive elements, utilizing tools such as RepeatMasker (v4.0.7; −species arabidopsis -a) and RepeatProteinMask (v4.0.7) [49] for sequence delineation and categorization. Concurrently, *de novo* predictions were facilitated by RepeatModeler (v1.0.4) and LTRharvest (v2.9.0; −similar 85 -mintsd 4 -maxtsd 20) [50], establishing a unique library of repetitive sequences, subsequently analyzed with RepeatMasker. Additionally, Tandem Repeats Finder (v4.09; trf Ar.fasta 2 7 7 80 10 50 500 -f -d -m) [51] was employed to detect tandem repeat sequences within the genome. Comparison of annotations across specific regions of interest was performed using bedtools (v2.30.0) [52].

To unveil the intricate gene distribution and structural dynamics within the genome, we amalgamated three predictive methodologies: homology-based, *de novo*, and transcriptome-assisted predictions, thereby enabling a comprehensive forecast of gene architecture. For homology-based predictions, annotation data from *Arabidopsis thaliana*, *Nepeta cataria* [5], *Perilla citriodora*, *Perilla frutescens* [31], *Salvia hispanica* [53], and *Tectona grandis* [54] were assimilated. Initially, the GeMoMa (v1.9; −GeMoMa.Score = ReAlign) [55] software was employed for homology-based gene prediction, followed by the utilization of structurally sound genes from these outcomes to refine the *de novo* prediction capabilities of software such as Augustus (v3.2.1; −species = arabidopsis) [56] and SNAP (v190514) [57], significantly enhancing the precision of our genomic analyses. For the treatment of RNA-Seq data, HISAT2 (v2.1.0) [58] was leveraged to align transcriptomic data against the genome, succeeded by the assembly of transcripts via StringTie (v2.2.1) [59]. Subsequent to this, open reading frames (ORFs) were delineated employing TransDecoder (v5.5.0). Integration of all derived data sets was accomplished using the Evidence Modeler [60] software, culminating in the formulation of the definitive gene set.

Post acquisition of structural gene data, our focus pivoted towards elucidating the functional attributes of the genes. Functional annotation of the gene set, extrapolated from gene structural annotations, was executed utilizing the Diamond alignment tool (v2.0.15) [61] against established protein databases, with functional domains being delineated via InterProScan [62]. The databases harnessed for this functional annotation included SwissProt [63], TrEMBL [63], KEGG [64], InterPro [62], NR, KOG, and GO [65], ensuring a comprehensive and multifaceted understanding of gene functionalities.

### RNA sequencing and gene expression analysis

In this study, samples comprising young leaves, old leaves, flowers, and stems of *Agastache rugosa* were systematically harvested, with each type of organ represented by three biological replicates. Total RNA was extracted from these samples using the CTAB method, followed by purification with oligo(dT) magnetic beads. The purified RNA was fragmented and reverse-transcribed into cDNA using random primers. Subsequently, the cDNA underwent end-repair and adaptor ligation, followed by PCR amplification of the ligated products. The amplified products were circularized to form a single-stranded DNA library, which was then prepared for high-throughput sequencing.

Quality control of the raw sequencing data was performed using SOAPnuke software (v1.5.6; −n 0.05 -l 5 -q 0.5 -Q 2) [66], which removed low-quality, adaptor-contaminated, and high-unknown-base-content reads, resulting in high-quality (clean) reads. These clean reads were aligned to the reference genome with HISAT2 (v2.1.0) [58], facilitating the prediction of novel transcripts. Transcripts demonstrating coding potential were integrated into the reference sequence, and gene expression levels were quantified using RSEM (v1.3.1; −forward-prob 0.5 −paired-end) [67], measured in Fragments Per Kilobase of transcript per Million mapped reads (FPKM). Differential gene expression analysis across various samples was conducted with DESeq2 (v1.4.5) [68]. The differentially expressed genes were subsequently analyzed for enrichment in GO [65] and KEGG [64] pathways using Phyper.

### Integration and analysis of hi-C data in *Agastache rugosa* genome

The previously acquired and rigorously quality-controlled Hi-C data will be employed to develop contact matrices specific to the *Agastache rugosa* genome, utilizing JuicerTools (v1.9.9) and Juicebox (v2.20) [45]. This approach will enable a detailed assessment of the genomic spatial organization. Building upon this foundational data, HiCExplorer (v3.7.2) [69] was utilized for further analytical endeavors, specifically to delineate TADs by the hicFindTADs (−correctForMultipleTesting fdr). Compartments A/B and loop structures are evaluated by arrowhead and hiccups, respectively. The identification of A/B compartments is performed at a resolution of 100 kb, whereas the identification of TADs and loops is executed at a resolution of 10 kb. The integration and visualization of complex genomic data, specifically within the 28.97 Mb to 32 Mb segment on chromosome 8, which includes RNA expression levels, genomic annotations, A/B compartments, TADs, and loops, were adeptly conducted using the pyGenomeTracks tool (v3.8). We employed the hicPlotViewpoint feature in HiCExplorer to explore the interactions between our target region (29.1 Mb–29.7 Mb) and all genes within Chr 8.

For interchromosomal interactions at 50 kbp resolution, intensities from across the BGC target region (29.1 Mb–29.7 Mb) were plotted across the lengths of chromosome. Normalization was achieved by removing zero intensity values, taking the log (base 2) of intensity values, and calculating z-scores across each chromosome. P-values were calculated from z-scores based proportion of a normally distributed is expected above the intensity value (pnorm function in R). For chromosome wide analyses p-values were corrected with Benjamini-Hochberg multiple test correction.

### Species tree and synteny

Whole-genome based phylogeny of mint family species was inferred using OrthoFinder (v2.5.4). Whole-genome sequences from various mint family (Lamiaceae) species were retrieved from public databases such as NCBI and other genomic repositories. Sequence alignments were performed using MAFFT (v7.450) [70] with default parameters. Orthologous groups were identified using the default parameters of OrthoFinder [33], which include using DIAMOND (v2.1.9) for sequence similarity searches in sensitive mode (−fast) and the default inflation value for MCL clustering. Species tree inference was performed using the STAG and STRIDE algorithms within OrthoFinder with default settings. Numbers on the branches indicate support values as determined by OrthoFinder. *Artemisia annua* was used as an outgroup to root the phylogenetic tree. The resulting phylogenetic tree was visualized using FigTree (v1.4.4; http://tree.bio.ed.ac.uk/software/figtree/) for clear presentation and interpretation.

### Gene trees

Genes for phylogenetics were selected from our previous work on *S. tenuifolia* [24]. Codon alignments of GOIs were performed using MAFFT (v7.450) with default settings. Maximum likelihood trees were inferred using IQ-Tree 2 (v2.2.3) [71] with ModelFinder [72], ultrafast bootstraps (UFBoot2, X1000) [73], and SH-aLRT supports (X1000) [74]. Gene trees were visualized using iTOL (v6.3.2) [75].

### Synteny analysis

The syntenic blocks were identified using JCVI (v1.2.7, python v3.9) [76]. Macrosyntenic blocks and Microsyntenic regions were identified using the default settings. Following the detection of synteny blocks, the data were visualized by MCscan (−iter = 1).

### Gene cloning and enzyme expression

Young leaf of *Agastache rugosa* was frozen in liquid nitrogen for RNA extraction by using the FastPure Plant Total RNA Isolation Kit (Polysaccharides and Polyphenolics-rich) (Vazyme Biotech, Nanjing, China). According to kit instructions., a cDNA library was constructed by using the HiScript III 1st Strand cDNA Synthesis Kit (+gDNA wiper) (Vazyme Biotech, Nanjing, China). Single-stranded cDNA was used as a template for PCR amplification with 2× Rapid Taq Master Mix (Vazyme Biotech, Nanjing, China) and gene-specific primers from the transcriptome database. The PCR products were separated and purified using the Trelief® DNA Gel Extraction Kit (Safe & Convenient) (Tsingke, Beijing, China) before cloning the DNA fragments into the pET28a vector (Vazyme Biotech) for sequencing. Details of the primers and vectors required for cloning and expression are provided in Supplementary Table S10. All genes were verified by Sanger Sequencing (Sangon Biotech, Shanghai, China).

The positive recombined vectors were transferred into BL21(DE3) by heat-shock. The positive single colony was inoculated into 5 ml of LB medium with 50 ng/ml kanamycin, then transferred to 100 ml of the same medium for further incubation at 37°C until the OD_600_ reached 1.0. Protein expression was induced by adding isopropyl β-D-1-thiogalactopyranoside to a final concentration of 1 mM, followed by incubation for 24 hours. The induction temperature was listed in Supplementary table 1. The cells were harvested by centrifugation at 5000 rpm for 15 min at 4°C and resuspended in 5 ml of lysis buffer (10 mM Tris–HCl [pH 8.0], 200 mM NaCl, 5% glycerol). The suspension was subjected to sonication, with 2 s of sonication followed by a 3 s interval on ice, repeating the process as needed. After centrifugation at 12 000 rpm for 30 min at 4°C, the supernatant was collected as crude protein extract.

### Enzyme assays in *vitro* and GC–MS analysis

For the NADPH-dependent reduction reaction (IPR), the reaction system (0.4 ml) consisted of buffer B (50 mM KH₂PO₄, 10% sorbitol, 1 mM DTT [pH 7.5]), containing 20 μM substrate, 10 mM NADPH tetrasodium salt hydrate, 6 mM glucose-6-phosphate, 20 U glucose-6-phosphate dehydrogenase, and 50 μl of protein extract. For the NAD-dependent reduction reaction (ISPD), NADPH was replaced with 10 mM NAD. 200 μl n-hexane was added on the top of the reaction mixture. After gently stirring the reaction at 31°C for 16 h, the reaction vial was frozen at −80°C and the upper organic phase was transferred for GC–MS analysis [1].

The gas chromatography–mass spectrometry (GC–MS) analysis utilized an 5973 mass spectrometer (Agilent Technologies, Santa Clara, California, USA), along with an Agilent column (19091S-433-HP-5 ms, 30 m × 250 μm × 0.25 μm). Helium served as the carrier gas with a set flow rate of 1.2 ml/min, and the injector temperature was maintained at 220°C. The mass spectrometer operated in electron impact (EI) mode at 70 eV, with temperatures for the quadrupole detector, ion source, and transfer line set to 150°C, 230°C, and 280°C, respectively. Identification and quantification of compounds were performed using the selected ion monitoring (SIM) mode. For enzyme activity analysis, the GC–MS settings were as follows: an initial temperature of 50°C, held for 3 min, followed by a ramp of 3°C/min up to 90°C, and then a rate of 5°C/min up to 150°C. The injection volume was set at 1.0 μl, with a splitless injection mode.

## Supplementary Material

Web_Material_uhaf034

## Data Availability

The genome sequence and raw sequencing reads are available at National Genomics Data Center (BioProject PRJCA027417). The raw reads are available in the Genome Sequence Archive (PacBio and Hi-C: CRA017422; RNA-seq: CRA017425). Gene sequences for *A. rugosa* ISPD and IPRs can be found in accession GB0004888.
