# Supplementary material for: Haplotype-resolved genome of Agastache rugosa (Huo Xiang) provides insight into monoterpenoid biosynthesis and gene cluster evolution
Source: Hortic Res. 2025 Feb 1;12(5):uhaf034. doi: 10.1093/hr/uhaf034 (PMC11992331; doi:10.1093/hr/uhaf034)
Supplement: Web_Material_uhaf034 [file web_material_uhaf034.docx]

Supplementary Material for

[**Haplotype-resolved genome of**](https://academic.oup.com/hr/advance-article/doi/10.1093/hr/uhae103/7642244?searchresult=1) [***Agastache rugosa***](https://academic.oup.com/hr/advance-article/doi/10.1093/hr/uhae103/7642244?searchresult=1) **(Huo Xiang) provides insight into monoterpenoid biosynthesis and gene cluster evolution**

Chanchan Liu^1,2#^*, DiShuai Li^1,2#^, Jingjie Dang^1,2^, Juan Shu^1,2^, Samuel J. Smit^3^, QiNan Wu^1,2^

and Benjamin R. Lichman^3^*

**Supplementary Tables**

**Table S1. HiFi sequencing data statistics for *Agastache rugosa*.**

| **Cell** | **Clean reads** | **Clean base (Gb)** | **Max length (bp)** | **Mean length (bp)** | **N50 (bp)** |
| --- | --- | --- | --- | --- | --- |
| m64061_220319_103826 | 1,897,472 | 31.82 | 49,940 | 16769.69 | 16,629 |

**Table S2. Hi-C sequencing data statistics for *Agastache rugosa*.**

| **Lane** | **Read length (bp)** | **Raw base (Gb)** | **Clean base (Gb)** |
| --- | --- | --- | --- |
| V350049616_L01_CWHPE22031998-81 | 150,150 | 16.6 | 16.47 |
| V350049616_L01_CWHPE22031998-82 | 150,150 | 22.03 | 21.86 |
| V350049616_L01_CWHPE22031998-83 | 150,150 | 17.51 | 17.37 |
| V350049616_L01_CWHPE22031998-84 | 150,150 | 19.97 | 19.81 |
| V350049616_L01_CWHPE22031998-85 | 150,150 | 12.6 | 12.5 |
| V350049616_L01_CWHPE22031998-86 | 150,150 | 19.59 | 19.43 |
| V350049616_L01_CWHPE22031998-87 | 150,150 | 15.35 | 15.22 |
| V350049616_L01_CWHPE22031998-88 | 150,150 | 15.62 | 15.49 |
| Total | - | 139.26 | 138.14 |

**Table S3. Chromosome horizontal assembly results statistics for assembled haplotypes.**

| **Sample** |  | **Contig** | | **Scaffold** | |
| --- | --- | --- | --- | --- | --- |
|  |  | **Size (bp)** | **Number** | **Size (bp)** | **Number** |
| **Hap1** | N10 | 80,154,169 | 1 | 80,154,169 | 1 |
|  | N20 | 58,343,894 | 2 | 60,312,361 | 2 |
|  | N30 | 56,519,819 | 3 | 57,835,692 | 3 |
|  | N40 | 56,519,819 | 3 | 57,835,692 | 3 |
|  | N50 | 43,089,470 | 5 | 56,519,819 | 4 |
|  | N60 | 30,736,666 | 6 | 46,120,855 | 5 |
|  | N70 | 24,082,414 | 8 | 43,603,486 | 6 |
|  | N80 | 17,971,804 | 10 | 36,227,727 | 8 |
|  | N90 | 12,380,725 | 13 | 32,248,885 | 9 |
|  | Max Length | 80,154,169 | - | 80,154,169 | - |
|  | Total Number | - | 758 | - | 694 |
|  | Total Length | 487,162,419 | - | 487,194,419 | - |
|  | GC Ratio | 0.37 | - | 0.37 | - |
| **Hap2** | N10 | 79,602,723 | 1 | 79,602,723 | 1 |
|  | N20 | 58,949,900 | 2 | 60,371,479 | 2 |
|  | N30 | 58,899,195 | 3 | 59,894,288 | 3 |
|  | N40 | 58,899,195 | 3 | 59,894,288 | 3 |
|  | N50 | 47,976,063 | 4 | 58,899,195 | 4 |
|  | N60 | 43,540,121 | 5 | 44,703,048 | 5 |
|  | N70 | 38,943,965 | 6 | 43,540,121 | 6 |
|  | N80 | 30,597,870 | 8 | 38,943,965 | 7 |
|  | N90 | 21,444,924 | 10 | 36,259,677 | 8 |
|  | Max Length | 79,602,723 | - | 79,602,723 | - |
|  | Total Number | - | 167 | - | 92 |
|  | Total Length | 468,104,839 | - | 468,142,339 | - |
|  | GC Ratio | 0.37 | - | 0.37 | - |

**Table S4. Chromosome length statistics for assembled haplotypes.**

| **Sample** | **Chromosome ID** | **Number of contig in chromosome** | **Size (bp)** |
| --- | --- | --- | --- |
| **Hap1** | Chr1.1 | 1 | 80,154,169 |
|  | Chr2.1 | 3 | 60,312,361 |
|  | Chr3.1 | 3 | 57,835,692 |
|  | Chr4.1 | 1 | 56,519,819 |
|  | Chr5.1 | 5 | 46,120,855 |
|  | Chr6.1 | 1 | 43,603,486 |
|  | Chr7.1 | 4 | 38,721,742 |
|  | Chr8.1 | 3 | 36,227,727 |
|  | Chr9.1 | 2 | 32,248,885 |
| **Hap2** | Chr1.2 | 1 | 79,602,723 |
|  | Chr2.2 | 2 | 59,894,288 |
|  | Chr3.2 | 2 | 60,371,479 |
|  | Chr4.2 | 1 | 58,899,195 |
|  | Chr5.2 | 4 | 44,703,048 |
|  | Chr6.2 | 1 | 43,540,121 |
|  | Chr7.2 | 1 | 38,943,965 |
|  | Chr8.2 | 1 | 36,259,677 |
|  | Chr9.2 | 2 | 32,146,750 |

**Table S5. BUSCO evaluation of haploid assembly results.** Complete BUSCOs (C): the number of genes fully covered; Complete and single-copy BUSCOs (S): the number of genes with complete coverage and single copy; Complete and duplicated BUSCOs (D): the number of genes that are completely duplicated and are multiple copies; Fragmented BUSCOs (F): the number of genes fragmented buscos, only partially fragmented; Missing BUSCOs (M): the number of missing genes.

| **Sample** | **Type** | **Contig level** | | **Chromosome level** | | **Annotation** | |
| --- | --- | --- | --- | --- | --- | --- | --- |
|  |  | **Number** | **Percentage (%)** | **Number** | **Percentage (%)** | **Number** | **Percentage (%)** |
| **Hap1** | Complete BUSCOs (C) | 2,263 | 97.29 | 2,263 | 97.29 | 2,228 | 95.79 |
|  | Complete and single-copy BUSCOs (S) | 2,175 | 93.51 | 2,175 | 93.51 | 2,132 | 91.66 |
|  | Complete and duplicated BUSCOs (D) | 88 | 3.78 | 88 | 3.78 | 96 | 4.13 |
|  | Fragmented BUSCOs (F) | 13 | 0.56 | 13 | 0.56 | 15 | 0.64 |
|  | Missing BUSCOs (M) | 50 | 2.15 | 50 | 2.15 | 83 | 3.57 |
|  | Total BUSCO groups searched | 2,326 | - | 2,326 | - | 2,326 | - |
| **Hap2** | Complete BUSCOs (C) | 2,271 | 97.64 | 2,271 | 97.64 | 2,245 | 96.52 |
|  | Complete and single-copy BUSCOs (S) | 2,183 | 93.85 | 2,183 | 93.85 | 2,155 | 92.65 |
|  | Complete and duplicated BUSCOs (D) | 88 | 3.78 | 88 | 3.78 | 90 | 3.87 |
|  | Fragmented BUSCOs (F) | 12 | 0.52 | 12 | 0.52 | 8 | 0.34 |
|  | Missing BUSCOs (M) | 43 | 1.85 | 43 | 1.85 | 73 | 3.14 |
|  | Total BUSCO groups searched | 2,326 | - | 2,326 | - | 2,326 | - |

**Table S6. Repeat sequence statistics.** TRF refers to tandem repeats identified in the genome sequence using the Tandem Repeats Finder software. RepeatMasker and RepeatProteinMask annotate transposable elements in the genome sequence based on the RepBase database, utilizing respectively the RepeatMasker and RepeatProteinMask software. *De novo* refers to the results obtained by annotating the genome sequence with RepeatMasker using a library of *de novo* transposable elements (TEs) predicted by LTRharvest and RepeatModeler. Total represents the non-redundant outcomes derived from all mentioned methods, excluding overlapping results.

| **Sample** | **Type** | **Repeat Length (bp)** | **Proportions in the genome (%)** |
| --- | --- | --- | --- |
| **Hap1** | LTRharvest | 74,186,128 | 15.2282 |
|  | denovo | 284,315,846 | 58.3616 |
|  | ProteinMask | 42,858,055 | 8.7975 |
|  | RepeatMasker | 41,715,558 | 8.563 |
|  | TRF | 52,393,278 | 10.7548 |
|  | Total | 301,731,873 | 61.9366 |
| **Hap2** | LTRharvest | 65,127,781 | 13.9131 |
|  | denovo | 280,318,699 | 59.8837 |
|  | ProteinMask | 43,422,529 | 9.2762 |
|  | RepeatMasker | 41,849,978 | 8.9403 |
|  | TRF | 52,736,729 | 11.266 |
|  | Total | 296,108,395 | 63.2569 |

**Table S7** Repeat sequence classification result statistics

| **Sample** | **Type** | | **Length (bp)** | **Proportions in the genome (%)** |
| --- | --- | --- | --- | --- |
| **Hap1** | Retro | LTR/Copia | 86,311,793 | 17.71725 |
|  |  | LTR/Gypsy | 88,893,335 | 18.24717 |
|  |  | LTR/Other | 122,295,452 | 25.10363 |
|  |  | SINE | 1,022,975 | 0.20999 |
|  |  | LINE | 1,877,182 | 0.38533 |
|  |  | Other | 0 | 0.0 |
|  | DNA | Academ | 100,818 | 0.02069 |
|  |  | CACTA | 1,807,313 | 0.37099 |
|  |  | Crypton | 96,889 | 0.01989 |
|  |  | Dada | 20,734 | 0.00426 |
|  |  | Ginger | 66,038 | 0.01356 |
|  |  | hAT | 3,018,789 | 0.61967 |
|  |  | Helitron | 912,487 | 0.18731 |
|  |  | Kolobok | 376,527 | 0.07729 |
|  |  | Mutator-like | 1,626,139 | 0.3338 |
|  |  | P_Element | 82,631 | 0.01696 |
|  |  | PIF-Harbinger | 809,778 | 0.16622 |
|  |  | PiggyBac | 233,962 | 0.04803 |
|  |  | Sola | 191,742 | 0.03936 |
|  |  | TcMar | 590,668 | 0.12125 |
|  |  | Other | 656,837 | 0.13483 |
|  | Other | - | 11,361 | 0.00233 |
|  | Unknown | - | 15,930,769 | 3.27011 |
|  | Total | - | 296,601,133 | 60.88342 |
| **Hap2** | Retro | LTR/Copia | 84,377,045 | 18.02525 |
|  |  | LTR/Gypsy | 88,777,181 | 18.96523 |
|  |  | LTR/Other | 118,939,358 | 25.4087 |
|  |  | SINE | 1,036,660 | 0.22146 |
|  |  | LINE | 1,676,939 | 0.35824 |
|  |  | Other | 0 | 0.0 |
|  | DNA | Academ | 40,188 | 0.00859 |
|  |  | CACTA | 1,724,917 | 0.36849 |
|  |  | Crypton | 97,510 | 0.02083 |
|  |  | Dada | 22,764 | 0.00486 |
|  |  | Ginger | 55,825 | 0.01193 |
|  |  | hAT | 2,559,058 | 0.54668 |
|  |  | Helitron | 908,896 | 0.19417 |
|  |  | Kolobok | 180,116 | 0.03848 |
|  |  | Mutator-like | 1,606,470 | 0.34319 |
|  |  | P_Element | 77,760 | 0.01661 |
|  |  | PIF-Harbinger | 803,232 | 0.17159 |
|  |  | PiggyBac | 245,498 | 0.05245 |
|  |  | Sola | 76,130 | 0.01626 |
|  |  | TcMar | 489,260 | 0.10452 |
|  |  | Other | 601,444 | 0.12848 |
|  | Other | - | 9,336 | 0.00199 |
|  | Unknown | - | 11,317,326 | 2.41769 |
|  | Total | - | 291,140,914 | 62.19566 |

**Table S8** **Statistical results of basic structure of gene prediction**

| **Sample** | **Type** | **Gene set** | **Number** | **Average gene**  **Length (bp)** | **Average CDS**  **Length (bp)** | **Average exon**  **per gene** | **Average exon**  **Length (bp)** | **Average intron**  **Length (bp)** |
| --- | --- | --- | --- | --- | --- | --- | --- | --- |
| **Hap1** | *De novo* | Augustus | 87,284 | 2645.49 | 1380.65 | 4.06 | 340.28 | 413.69 |
|  |  | SNAP | 144,951 | 1671.95 | 882.08 | 3.67 | 240.64 | 296.32 |
|  | homolog | *A.thaliana* | 21,897 | 3036.28 | 1300.98 | 5.59 | 232.76 | 378.12 |
|  |  | *N.cataria* | 27,153 | 2814.0 | 1258.69 | 5.24 | 240.42 | 367.22 |
|  |  | *P.citriodora* | 24,902 | 2935.49 | 1304.7 | 5.19 | 251.44 | 389.32 |
|  |  | *P.frutescens* | 26,140 | 2925.33 | 1291.17 | 5.16 | 249.99 | 392.37 |
|  |  | *S.hispanica* | 23,756 | 3243.27 | 1259.7 | 5.6 | 224.77 | 430.8 |
|  |  | *T.grandis* | 27,568 | 2882.56 | 1273.91 | 5.14 | 247.87 | 388.61 |
|  | RNA-seq | AR-F-1A | 20,111 | 4018.52 | 1155.48 | 5.04 | 229.29 | 708.76 |
|  |  | AR-F-2A | 20,871 | 3959.0 | 1134.76 | 4.93 | 230.3 | 719.12 |
|  |  | AR-F-3A | 20,070 | 3821.19 | 1099.84 | 4.87 | 225.69 | 702.59 |
|  |  | AR-O-1A | 18,104 | 3934.49 | 1074.07 | 4.82 | 222.61 | 747.86 |
|  |  | AR-O-2A | 18,137 | 3778.13 | 1040.32 | 4.75 | 218.83 | 729.32 |
|  |  | AR-O-3A | 17,493 | 3804.48 | 1056.24 | 4.83 | 218.55 | 717.01 |
|  |  | AR-S-1A | 18,857 | 3962.37 | 1148.04 | 5.02 | 228.78 | 700.4 |
|  |  | AR-S-2A | 18,860 | 3960.53 | 1129.92 | 4.98 | 227.09 | 711.99 |
|  |  | AR-S-3A | 19,696 | 4072.66 | 1161.2 | 5.0 | 232.38 | 728.43 |
|  |  | AR-Y-1A | 19,152 | 4062.55 | 1151.91 | 5.0 | 230.61 | 728.55 |
|  |  | AR-Y-2A | 17,223 | 3783.28 | 1061.76 | 4.83 | 219.84 | 710.66 |
|  |  | AR-Y-3A | 18,093 | 3920.77 | 1086.8 | 4.88 | 222.93 | 731.32 |
|  | Final | - | 31,334 | 2898.48 | 1272.17 | 5.07 | 250.78 | 399.31 |
| **Hap2** | *De novo* | Augustus | 84,896 | 2633.04 | 1402.33 | 4.05 | 346.1 | 403.27 |
|  |  | SNAP | 138,457 | 1696.8 | 904.7 | 3.69 | 244.92 | 294.05 |
|  | homolog | *A.thaliana* | 22,206 | 3040.45 | 1308.38 | 5.6 | 233.65 | 376.55 |
|  |  | *N.cataria* | 27,659 | 2809.85 | 1257.52 | 5.24 | 240.2 | 366.52 |
|  |  | *P.citriodora* | 25,255 | 2938.02 | 1306.39 | 5.2 | 251.05 | 25,255 |
|  |  | *P.frutescens* | 26.549 | 2927.99 | 1293.15 | 5.18 | 249.88 | 26.549 |
|  |  | *S.hispanica* | 24,206 | 3232.85 | 1264.01 | 5.62 | 224.88 | 24,206 |
|  |  | *T.grandis* | 28,544 | 2884.71 | 1276.09 | 5.17 | 246.73 | 28,544 |
|  | RNA-seq | AR-F-1A | 23,651 | 4026.74 | 1171.09 | 5.09 | 230.25 | 23,651 |
|  |  | AR-F-2A | 24,516 | 3954.51 | 1150.42 | 4.99 | 230.65 | 24,516 |
|  |  | AR-F-3A | 23,755 | 3812.59 | 1114.88 | 4.92 | 226.72 | 23,755 |
|  |  | AR-O-1A | 21,095 | 3898.89 | 1090.37 | 4.89 | 223.0 | 21,095 |
|  |  | AR-O-2A | 20,703 | 3769.58 | 1075.48 | 4.9 | 219.64 | 20,703 |
|  |  | AR-O-3A | 20,577 | 3758.96 | 1071.98 | 4.87 | 220.01 | 20,577 |
|  |  | AR-S-1A | 22,294 | 3986.35 | 1166.94 | 5.05 | 231.09 | 22,294 |
|  |  | AR-S-2A | 22,326 | 3956.35 | 1147.69 | 5.02 | 228.56 | 22,326 |
|  |  | AR-S-3A | 23,130 | 4081.21 | 1177.19 | 5.02 | 234.41 | 23,130 |
|  |  | AR-Y-1A | 22,435 | 4064.94 | 1174.9 | 5.06 | 232.34 | 22,435 |
|  |  | AR-Y-2A | 20,008 | 3781.31 | 1075.81 | 4.92 | 218.66 | 20,008 |
|  |  | AR-Y-3A | 21,362 | 3921.04 | 1105.12 | 4.96 | 222.8 | 21,362 |
|  | Final | - | 31,578 | 2846.1 | 1261.66 | 5.01 | 252.07 | 31578 |

**Table S9. Annotations in specific genomics regions.** Proportion of bases annotated. Calculated using bedtools using whole genome including non-chromosomal scaffolds. Regions are specified by “chromosome:start-end” where start-end values are in kbp. Chromosome 8 peak regions were chosen manually as show in Figure S9 (22750-23000, 23900-24100, 25350-25450, 27750-27900, 314000-31500). Significant inter-chromosomal region interactions shown in Figure S10 (1:16900-16950, 3:6200-6250, 3:6550 6600, 3:10550-10600, 3:26750-26800, 3:57850-57900, 4:2800-2850, 4:18450-18500, 4:26500-26550, 4:3250-3300 and 4:12450-12500).

| GFF | Annotation | Genome | Full cluster | ISPD region | Core cluster | Chromosome 8 peaks | Significant interchromosomal | PR region |
| --- | --- | --- | --- | --- | --- | --- | --- | --- |
| Region | | All | 8:28980-29820 | 8:29100-29200 | 8:29450-29700 | Figure S9 | Figure S10 | 2:47000-47200 |
| Genes | mRNA | 0.19 | 0.28 | 0.17 | 0.15 | 0.20 | 0.17 | 0.07 |
| Genes | CDS | 0.08 | 0.09 | 0.08 | 0.06 | 0.07 | 0.08 | 0.02 |
| denovoRM | Transposon | 0.60 | 0.63 | 0.64 | 0.74 | 0.71 | 0.61 | 0.80 |
| denovoRM | LTR | 0.57 | 0.61 | 0.63 | 0.71 | 0.70 | 0.60 | 0.78 |
| denovoRM | LTR/unknown | 0.23 | 0.27 | 0.34 | 0.29 | 0.19 | 0.26 | 0.08 |
| denovoRM | LTR/Gypsy | 0.18 | 0.13 | 0.18 | 0.25 | 0.19 | 0.14 | 0.59 |
| denovoRM | LTR/Gypsy/Athila | 0.03 | 0.03 | 0.00 | 0.10 | 0.05 | 0.03 | 0.11 |
| denovoRM | LTR/Gypsy/Tekay | 0.10 | 0.01 | 0.00 | 0.02 | 0.09 | 0.04 | 0.44 |
| denovoRM | LTR/Copia | 0.17 | 0.20 | 0.11 | 0.18 | 0.29 | 0.19 | 0.11 |
| denovoRM | LTR/Copia/SIRE | 0.14 | 0.16 | 0.10 | 0.13 | 0.23 | 0.12 | 0.11 |
| denovoRM | LTR/Copia/Tork | 0.01 | 0.01 | 0.00 | 0.03 | 0.02 | 0.02 | 0.00 |
| ProteinMasker | LTR | 0.09 | 0.11 | 0.07 | 0.16 | 0.17 | 0.12 | 0.24 |
| ProteinMasker | LTR/Gypsy | 0.05 | 0.05 | 0.06 | 0.08 | 0.07 | 0.06 | 0.21 |
| ProteinMasker | LTR/Copia | 0.04 | 0.05 | 0.01 | 0.08 | 0.10 | 0.05 | 0.03 |

**Supplemental Table S10.** Primers and vectors used for recombinant protein production.

| **Gene** | **Sequence (5’-3’)** | **Vector** | **Induction temperature** |
| --- | --- | --- | --- |
| ISPD | F: cagcaaatgggtcgcggatccATGGCAAGCATGGATTTGAAA | pET28a | 37 ℃ |
|  | R: ttgtcgacggagctcgaattcTTAATTTGATGGGTTCATGAATGG |  |  |
| IPR-1-1172 | F: cagcaaatgggtcgcggatccATGGCAGCAATGGCAGAAAC | pET28a | 16 ℃ |
|  | R: ttgtcgacggagctcgaattcCTAATTTGGTTTTTCAGCATCAGATT |  |  |
| IPR-2-1195 | F: cagcaaatgggtcgcggatccATGGTGGAAACGACGGCG | pET28a | 16 ℃ |
|  | R: ttgtcgacggagctcgaattcCTAATTTGGTTTTTCAGCATCAGATT |  |  |

**Supplementary Figures**


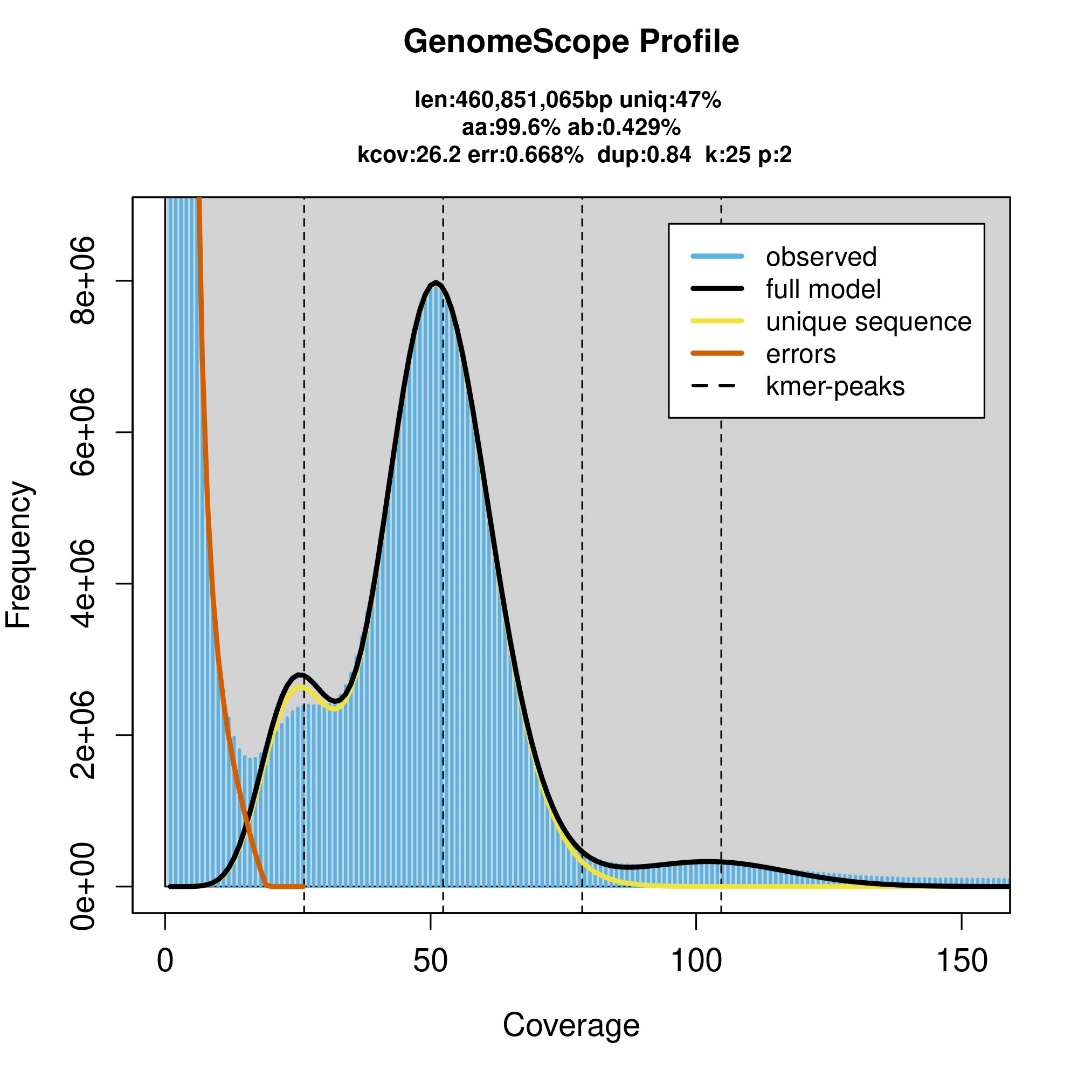


**Figure S1. Genomescope analysis of unassembled short reads.** Analysis on standard settings, using k-mer length of 25. Estimated genome size: 460,851 ,065 bp with 0.429% heterozygosity.


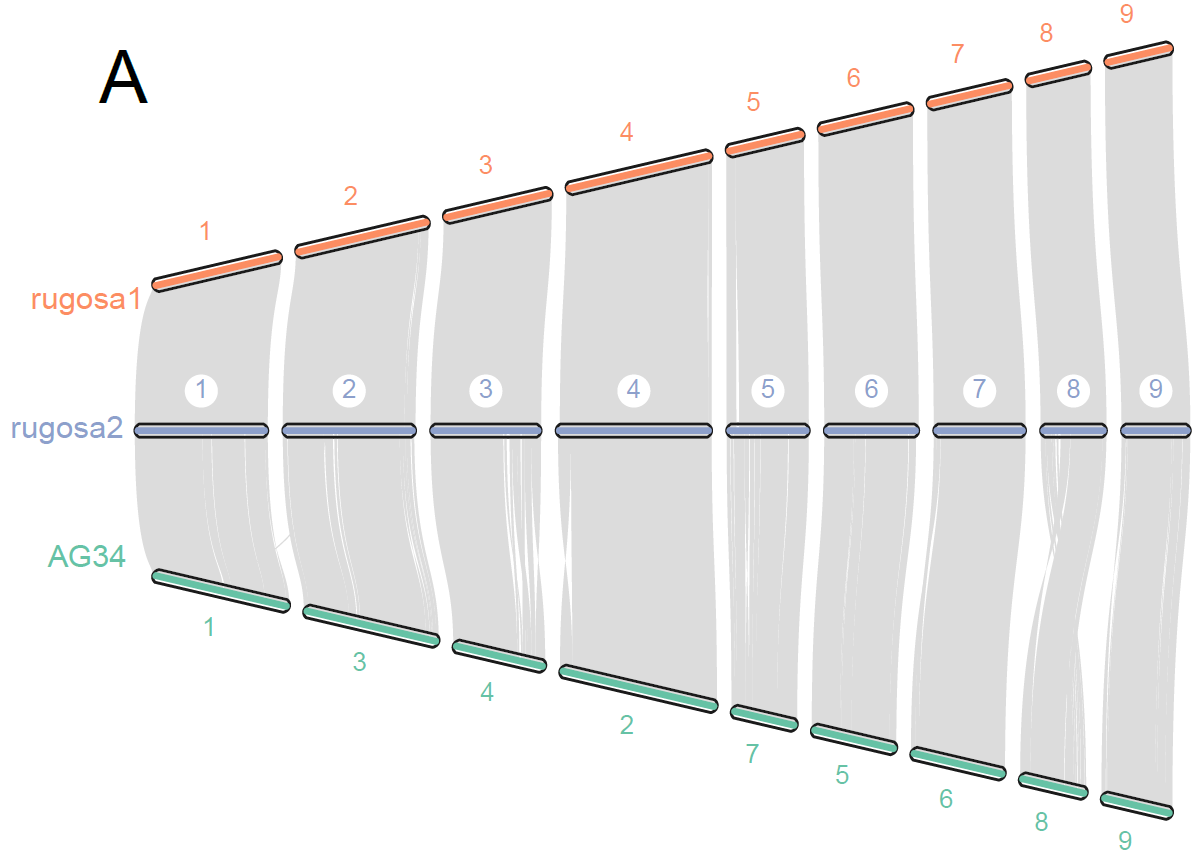

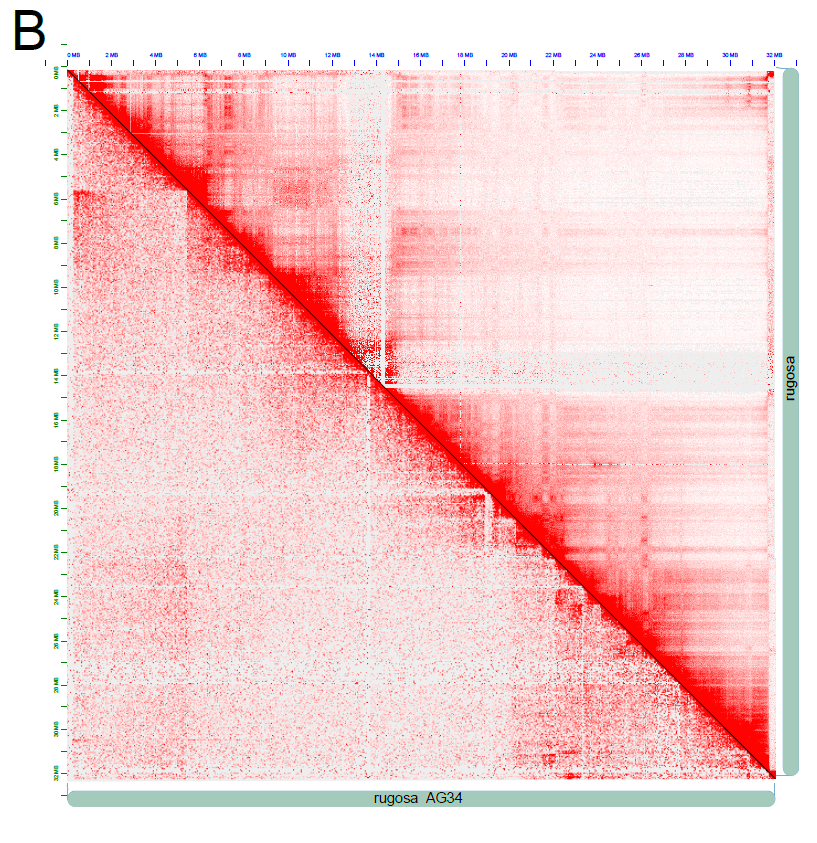


**Figure S2. Comparison of *Agastache rugosa* genome assemblies. A.** Macrosynteny comparison of the new haplotype resolved genome (rugosa1 and rugosa2 represent the different haplotypes) with the Park et al. genome (AG34) **B.** Comparison of Hi-C map of chromosome 8 of the two genomes highlighting difference in resolution.


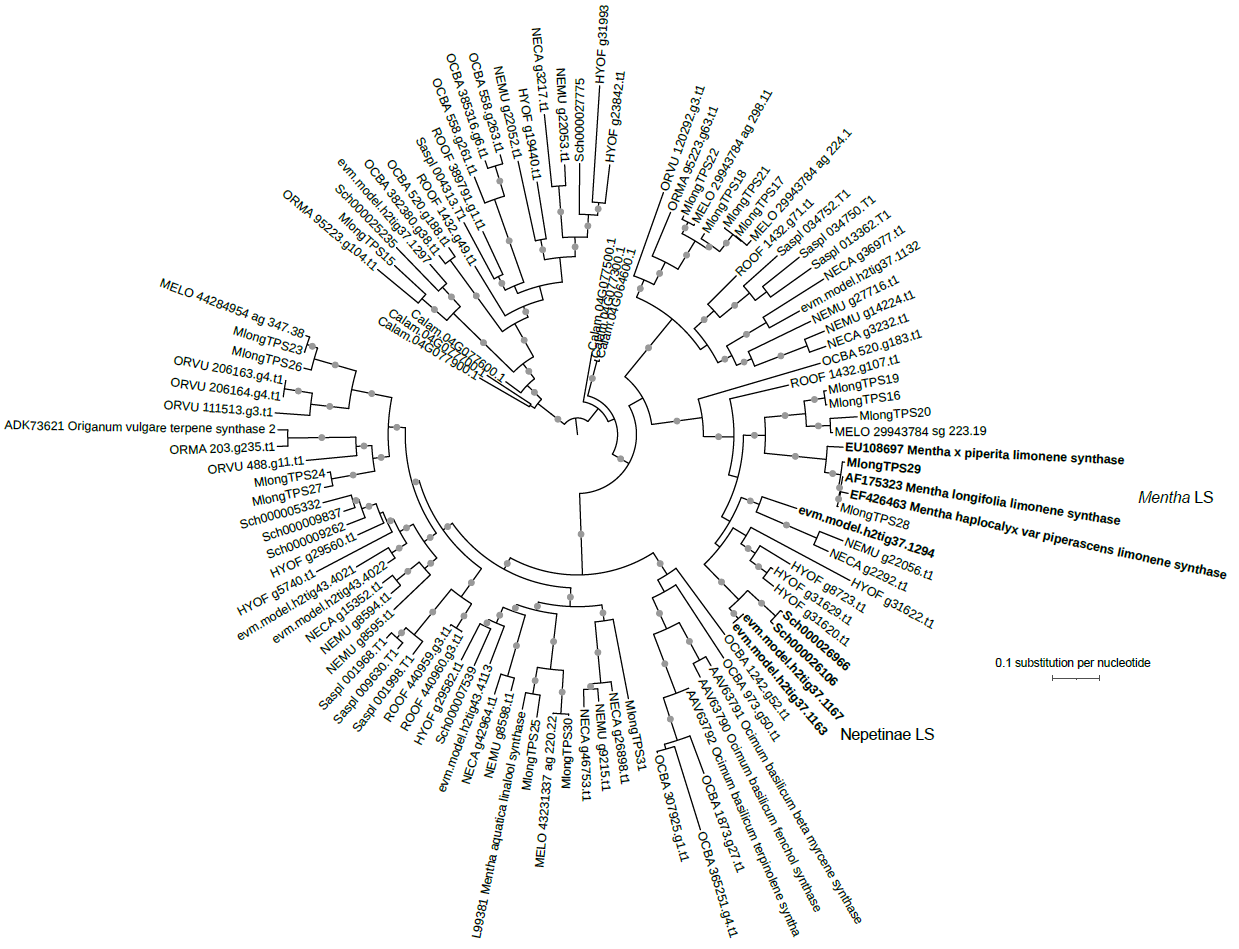


**Figure S3. Limonene synthase tree.** Limonene synthase maximum likelihood phylogenetic tree. Sequences of interest in bold: clustered LSs and TPS from *A. rugosa*, LS from *S. tenuifolia* and *Mentha* spp LS. Circles show branches with >85% and >95% support as judged by 1000X SH-aLRT and UltraFast Boostrapping replicates, respectively.

A


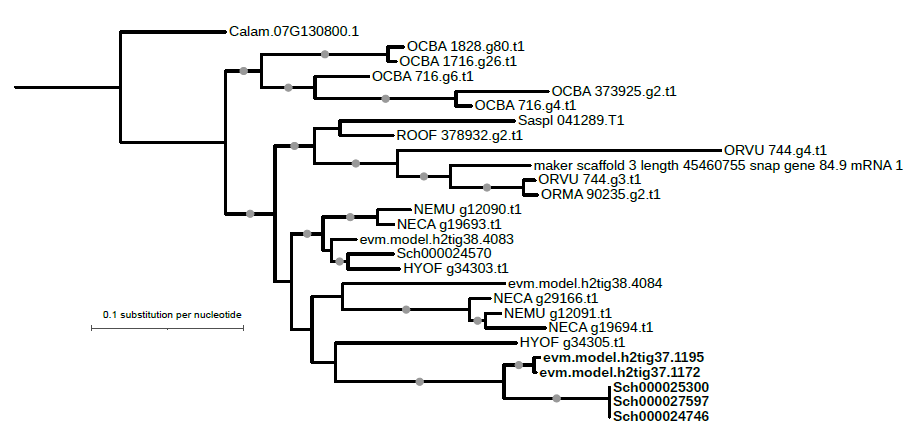


B
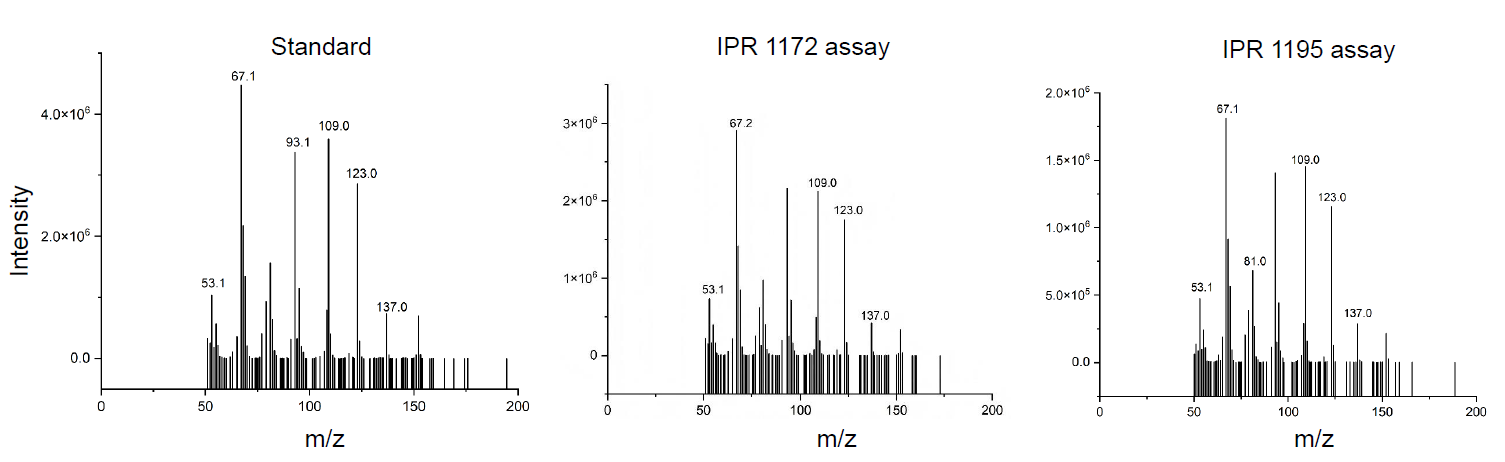


**Figure S4. Isopiperitenone reductases in *A. rugosa* A. Maximum likelihood phylogenetic tree of IPR sequences.** Circles show branches with >85% and >95% support as judged by 1000X SH-aLRT and UltraFast Boostrapping replicates, respectively. Genes of interest from *A. rugosa* and *S. tenuifolia* are in bold. **B.** **Isopulegone product validation.** EI spectra from isopulegone peak from standard (left), IPR-1172 assay (centre) and IPR-1195 assay (right) showing the same major fragments.


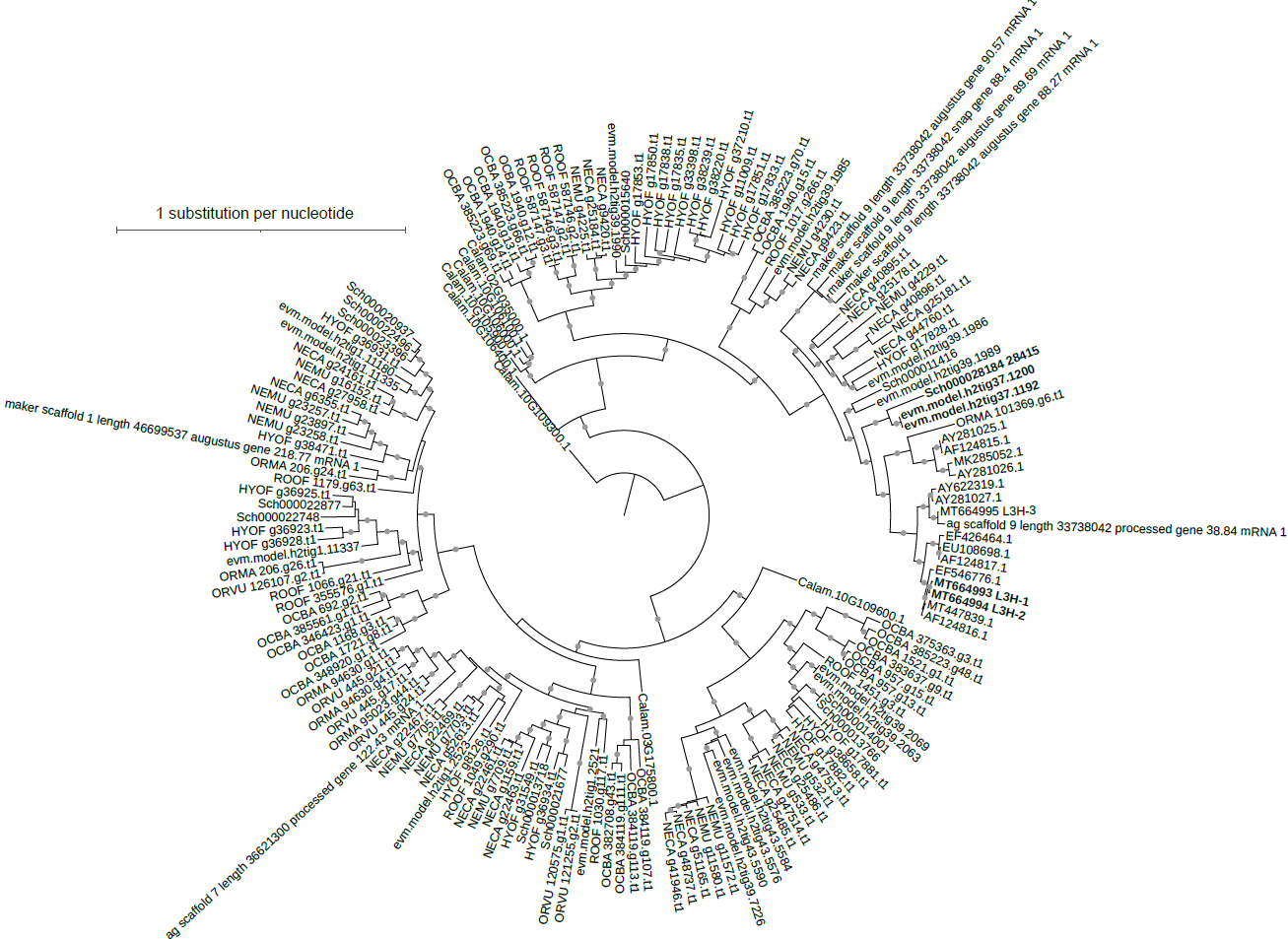


**Figure S5. Limonene 3-hydroxylase tree.** Limonene synthase maximum likelihood phylogenetic tree. Circles show branches with >85% and >95% support as judged by 1000X SH-aLRT and UltraFast Boostrapping replicates, respectively. Genes of interest from *A. rugosa*, *S. tenuifolia* and *M. longifolia* are in bold.

A


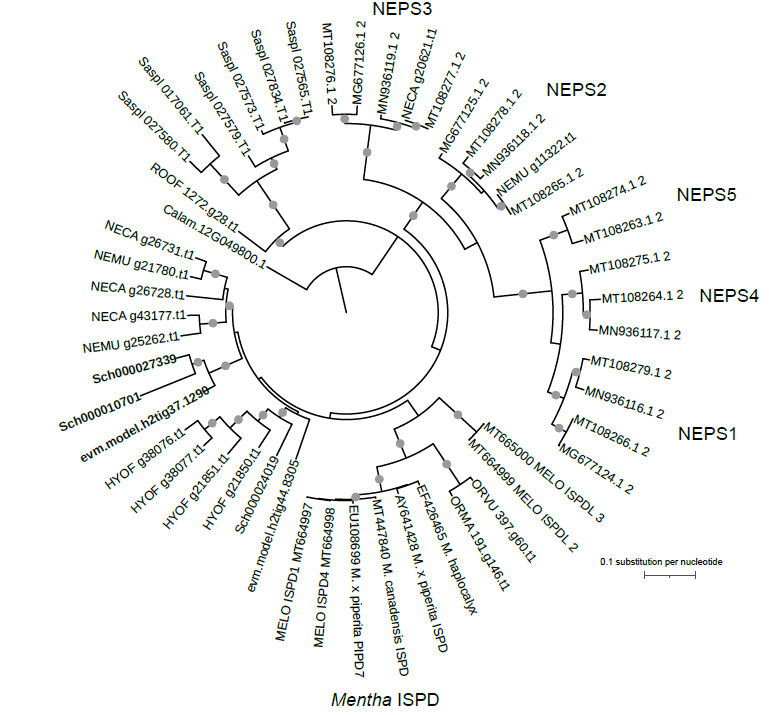


B
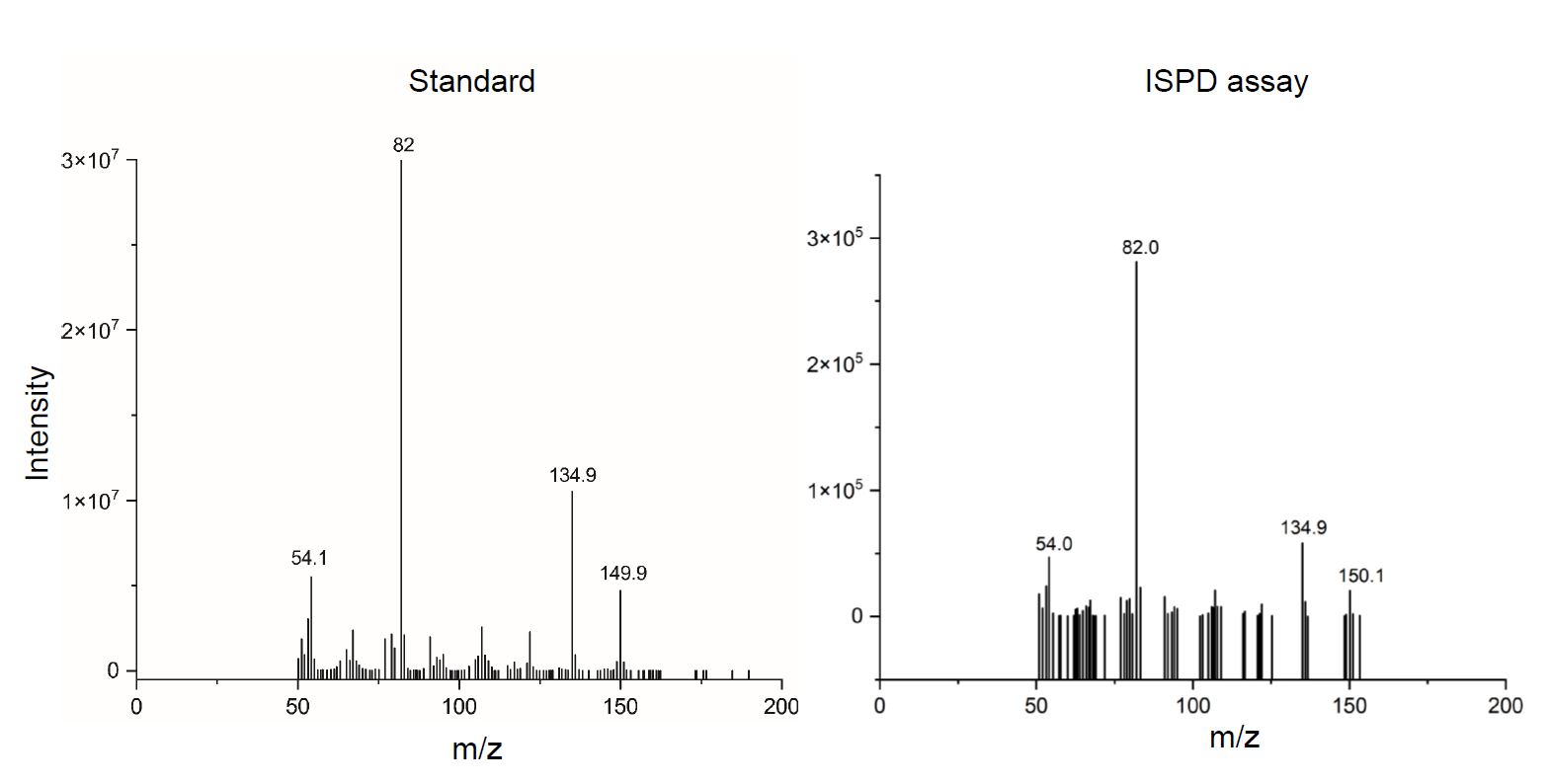


**Figure S6. Isopiperitenol dehydrogenases in *A. rugosa*.** **A.** Phylogenetic tree of isopiperitenol dehydrogenase (ISPD) including *Nepeta* spp NEPS and NEPS-like sequences. Circles show branches with >85% and >95% support as judged by 1000X SH-aLRT and UltraFast Boostrapping replicates, respectively. Genes of interest from *A. rugosa* and *S. tenuifolia* are in bold, other notable clades are annotated. **B.** **Isopiperitenone product validation.** EI spectra from isopiperitenone peak from standard (left) and ISPD assay (right), showing the same major fragments.


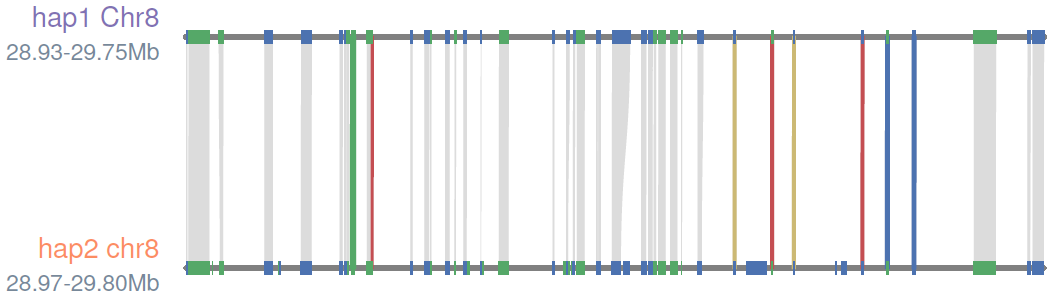


**Figure S7. Comparison of BGC region across two haplotypes.** Lines show orthologous genes with coloured lines highlighting biosynthetic genes.


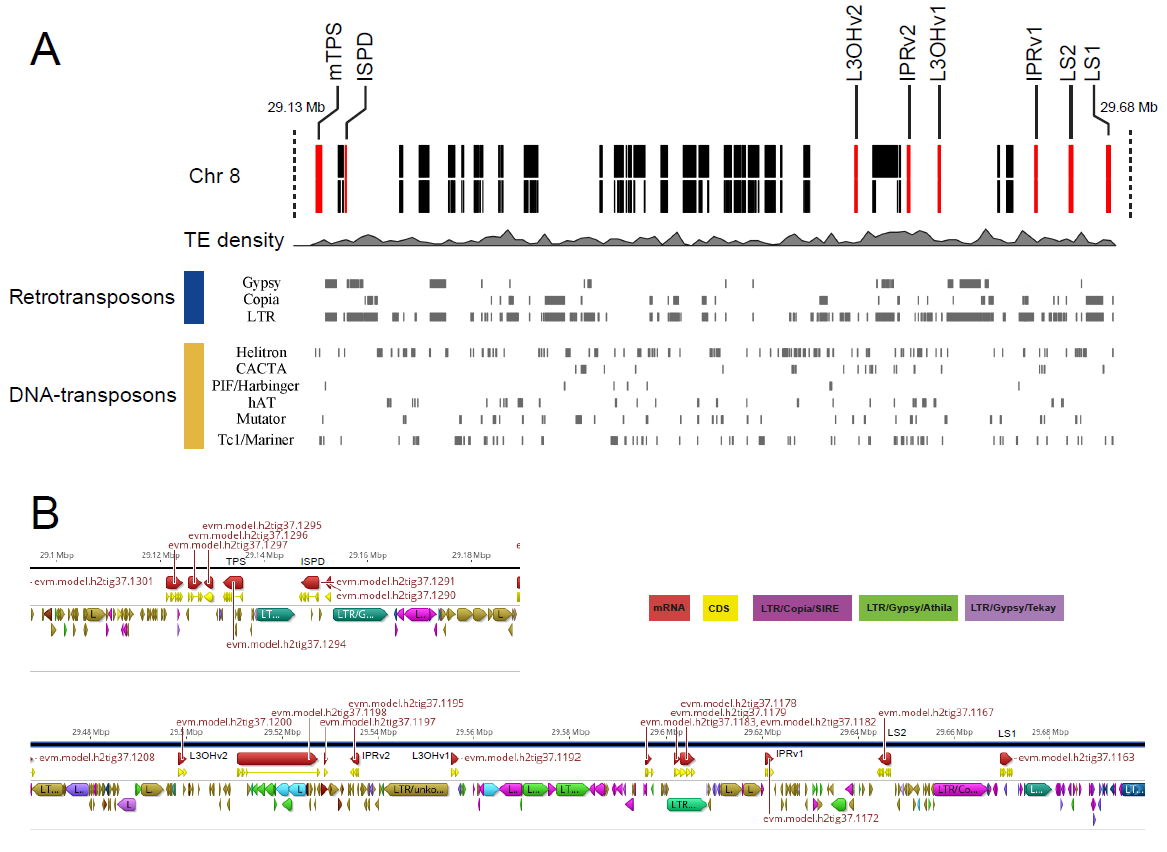


**Figure S8. Transposable element content of BGC. A.** Overview/density of TE content in the BGC, **B,** Detailed picture of TEs predicted from *de novo* TE RepeatMasker. Same TE class are coloured the same. Note TEs present within introns.


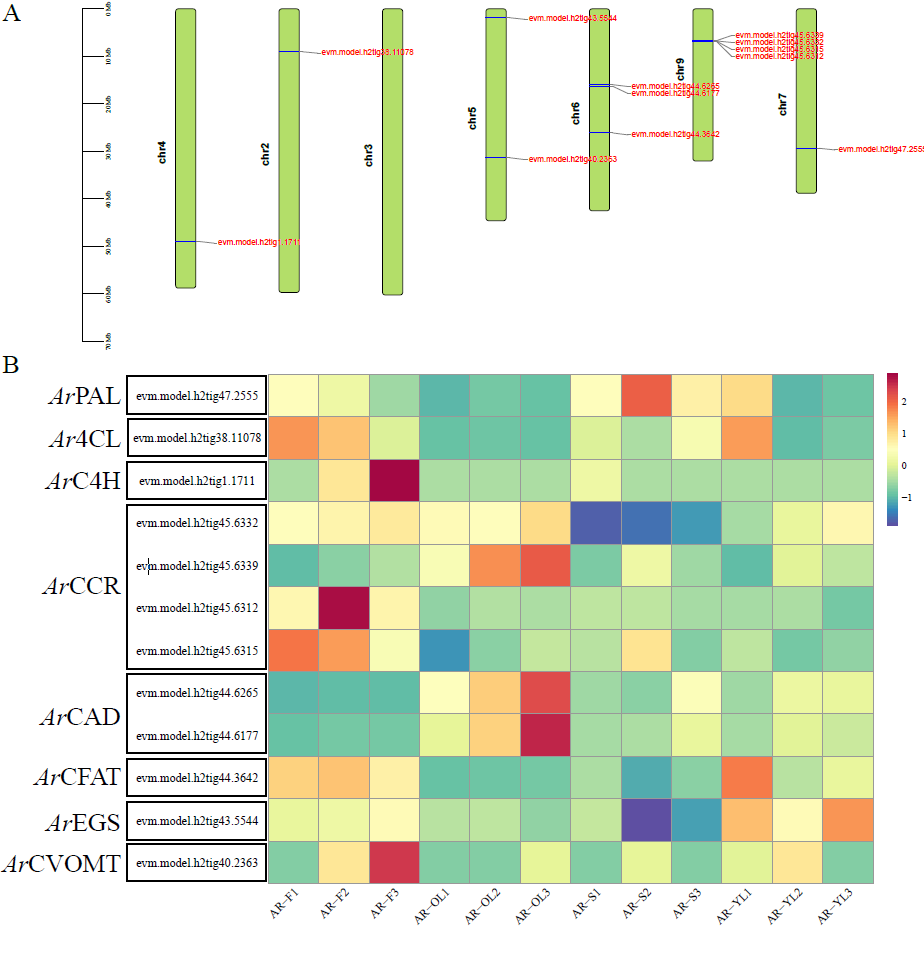


**Figure S9 Estragole genes. A.** Chromosomal location of genes involved in estragole biosynthesis. **B.** Expression matrix of estragole biosynthesis genes across different tissue types. RNAseq sample code (AR-Xn): where AR = *Agastache rugosa*, X = tissue type and n = biological repeat. Tissue types: F = flower, OL = old leaf, S = stem, YL = young leaf. Colors show z-score normalized expression. The estragole biosynthesis pathway: phenylalanine ammonia-lyase (PAL);4-coumarate:coenzyme A ligase (4CL); cinnamate 4-hydroxylase (4CH)[; cinnamoyl-CoA reductase (CCR); cinnamyl alcohol dehydrogenase (CAD); caffeoyl-CoA transferases (CFAT); eugenol synthase (EGS); chavicol O-methyl transferase (CVOMT).](http://html.rhhz.net/SWJSTB/html/2017-11-96.htm)


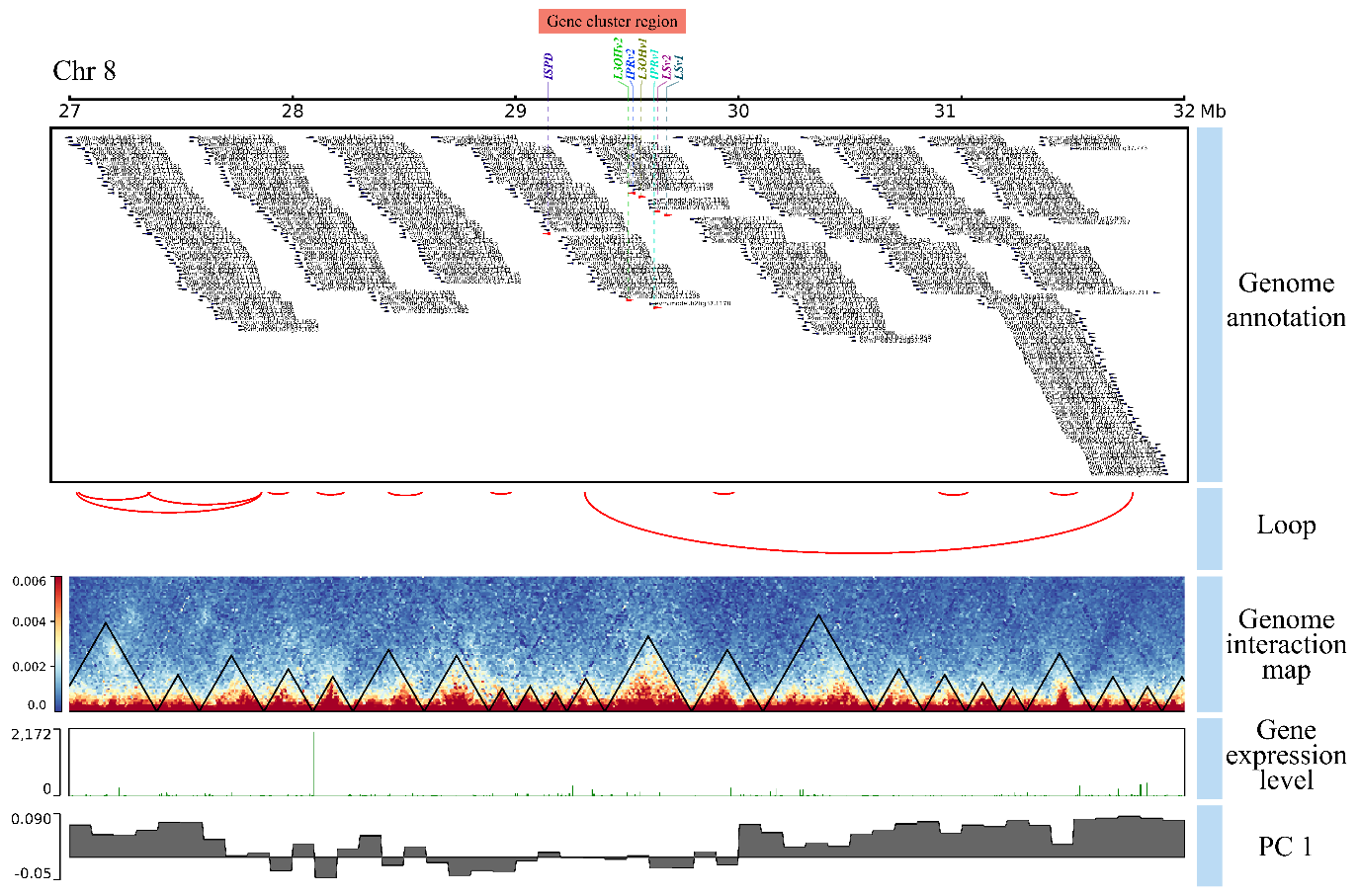


**Figure S10. Three-dimensional organisation of BGC.** Genomic annotations, loop structures, intergenic interaction heatmap, RNA-seq data, and PC1 values within the 27-32 Mb coordinates of chromosome 8.


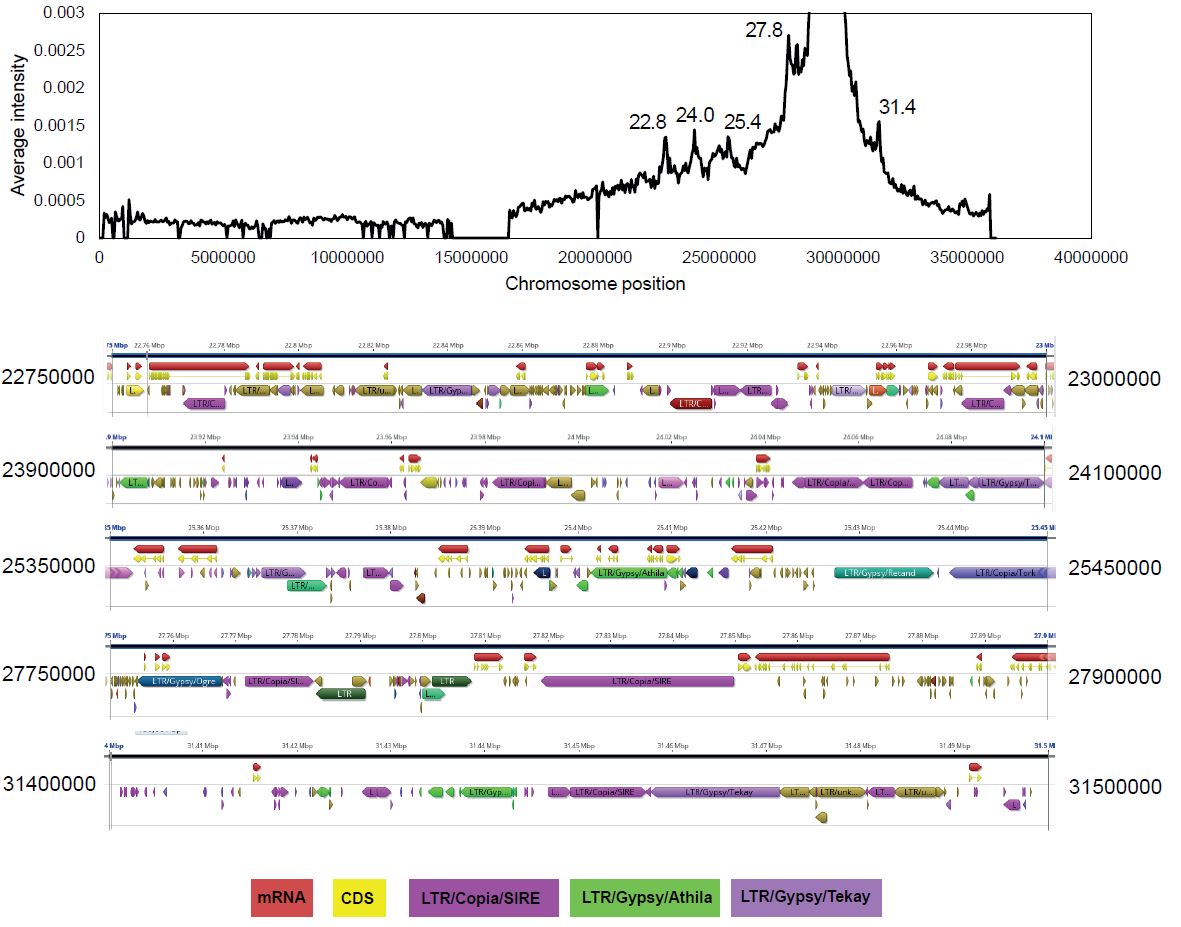


**Figure S11. Intrachromosomal interactions with the BGC.** Regions in chromosome 8 interacting with the whole BGC region (chromosome 8: 29.1-29.9 Mbp) as judged peaks in the average intensity of interactions. Annotations in the interacting regions shown below, with gene annotations and *de novo* RepeatMasker annotations depicted and colour coded.


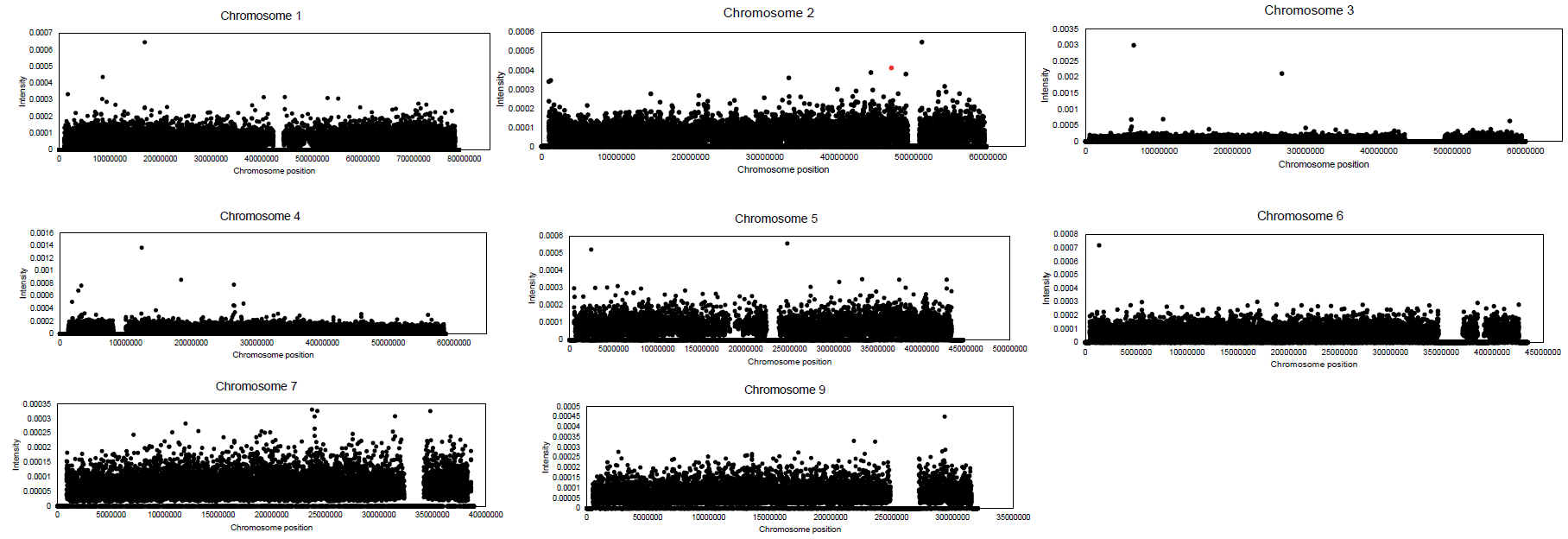


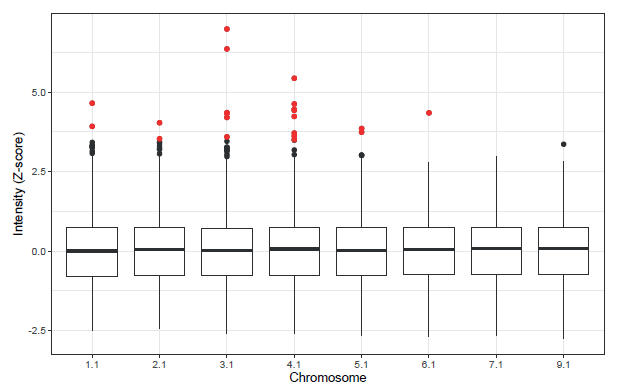


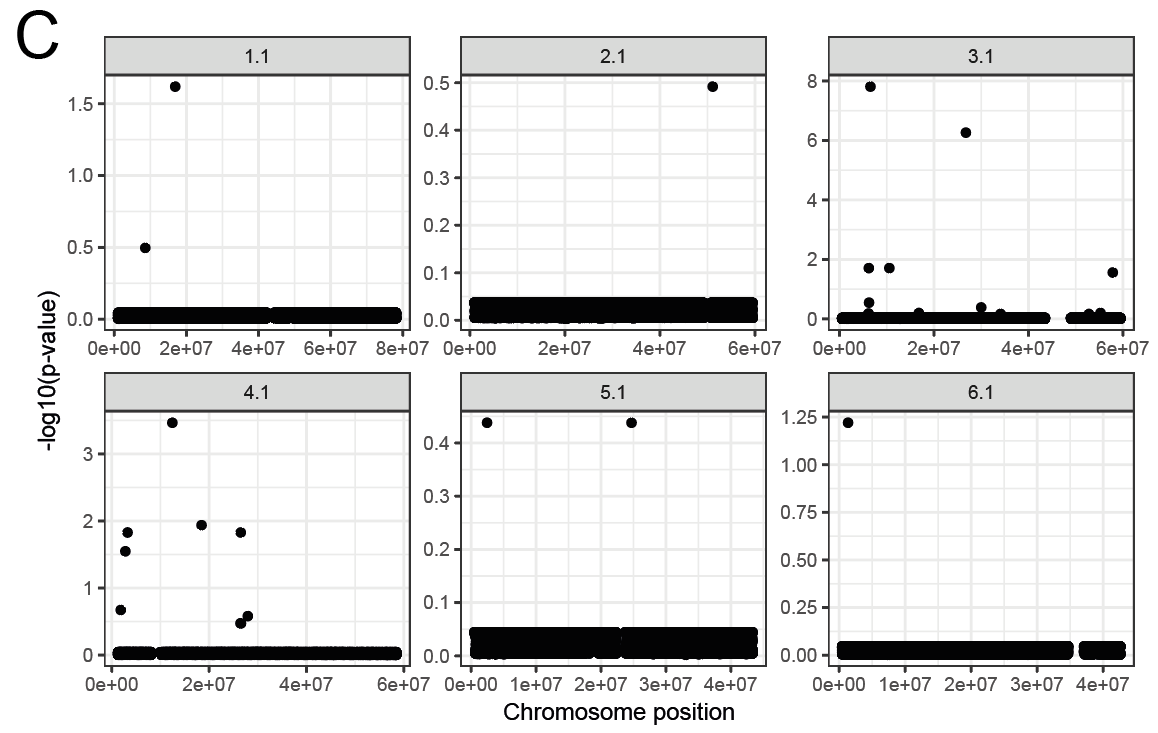


**Figure S12. Interchromosomal Hi-C interactions with the BGC. A**. Unnormalized intensities at 50 kbp resolution, from across the BGC target region (29.1 Mb-29.7 Mb). **B.** Intensities normalized across chromosomes and plotted as boxplots. Colored points are points greater than 3.5. **C.** Statistical significance of intensities from across chromosomes. P-values were calculated from z-scores based proportion of a normally distributed is expected above the intensity value. P-values were corrected with Benjamini-Hockberg multiple test correction and plotted as -log_10_ values wherein *p* = 0.05 is 1.3.

**References**

1 Liu C *et al.* A chromosome-level genome assembly reveals that a bipartite gene cluster formed via an inverted duplication controls monoterpenoid biosynthesis in Schizonepeta tenuifolia. *Mol Plant* 2023; **16**: 533–548.
